# Supplementary material for: CXCR3 enables recruitment and site-specific bystander activation of memory CD8+ T cells
Source: Nat Commun. 2019 Nov 1;10:4987. doi: 10.1038/s41467-019-12980-2 (PMC6825240; doi:10.1038/s41467-019-12980-2)
Supplement: Supplementary file 1 — Supplementary Information [file 41467_2019_12980_MOESM1_ESM.pdf]

CXCR3 enables recruitment and site-specific bystander activation of memory CD8 T cells

Maurice *et al.*

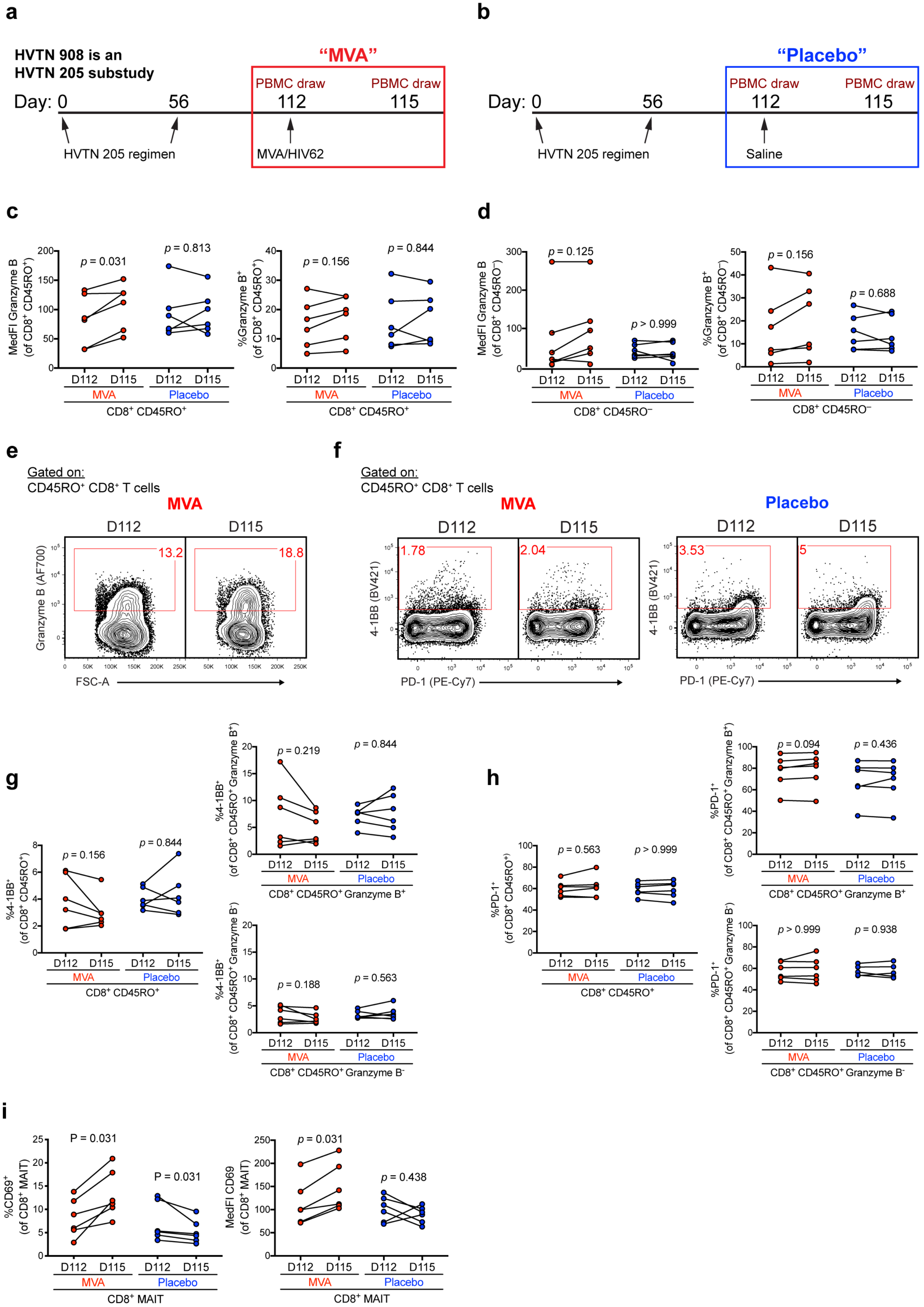
a
HVTN 908 is an
HVTN 205 substudy
"MVA"
PBMC draw
PBMC draw
Day: 0
56
112
115
HVTN 205 regimen
MVA/HIV62
b
"Placebo"
Saline
c
d
CD8⁺ CD45RO⁺
CD8⁺ CD45RO⁻
e
Gated on:
CD45RO⁺ CD8⁺ T cells
MVA
D112
D115
13.2
18.8
Granzyme B (AF700)
FSC-A
f
Placebo
1.78
2.04
3.53
5
4-1BB (BV421)
PD-1 (PE-Cy7)
g
h
CD8⁺ CD45RO⁺ Granzyme B⁺
CD8⁺ CD45RO⁺ Granzyme B⁻
i
CD8⁺ MAIT

Supplementary Fig. 1. Evidence for bystander-activation of human memory CD8$^+$ T cells following vaccination. **(a, b)** Immunization schematics for HVTN 908 “Early Immune Responses to Vaccination,” a substudy to HVTN 205 (see Methods). **(a)** In HVTN 908, some participants of the HVTN 205 trial received a boost with an MVA vector (day 112). We interrogated PBMC samples collected immediately prior to the MVA boost or saline placebo (day 112) and on day 3 post MVA-immunization (day 115) to determine if we could detect any memory CD8$^+$ T cells with a phenotype indicative of bystander-activation. **(b)** The HVTN 908 clinical trial design included a cohort receiving saline instead of the MVA vector and PBMCs from this placebo cohort were included in our sample analysis (we were blinded until flow cytometry data were acquired and analyzed). The samples from this placebo cohort served as an important control for potential batch effects between day 112 and day 115 samples to ensure that we could distinguish differences in biology from differences that may be technical in nature (for example, differences that could be introduced in sample processing, etc.). **(c-i)** Flow cytometric phenotyping of PBMC subsets in HVTN 908. **(c)** Granzyme B expression (median fluorescence intensity (MedFI) and frequency) in CD45RO$^+$ CD8$^+$ T cells. **(d)** Granzyme B MedFI and frequency in CD45RO$^-$ CD8$^+$ T cells. **(e)** Representative granzyme B staining in CD45RO$^+$ CD8$^+$ T cells. **(f)** Representative staining of PD-1 (x-axis) and 4-1BB (y-axis) in CD45RO$^+$ CD8$^+$ memory T cells. **(g)** 4-1BB or **(h)** PD-1 frequency of bulk, granzyme B$^+$, and granzyme B$^-$ CD45RO$^+$ CD8$^+$ memory T cells. **(i)** Frequency and MedFI of CD69 in CD8$^+$ MAIT cells. In **(c, d, g-i)** each symbol represents one donor at one timepoint (n=6 MVA vaccine recipients, n=6 placebo recipients). Connected symbols are from the same donor; all samples were stained and analyzed at once. Indicated are statistical significances by Wilcoxon matched-pairs signed rank test. Gating strategies are depicted in Supplementary Fig. 9. Source data are provided as a Source Data file.

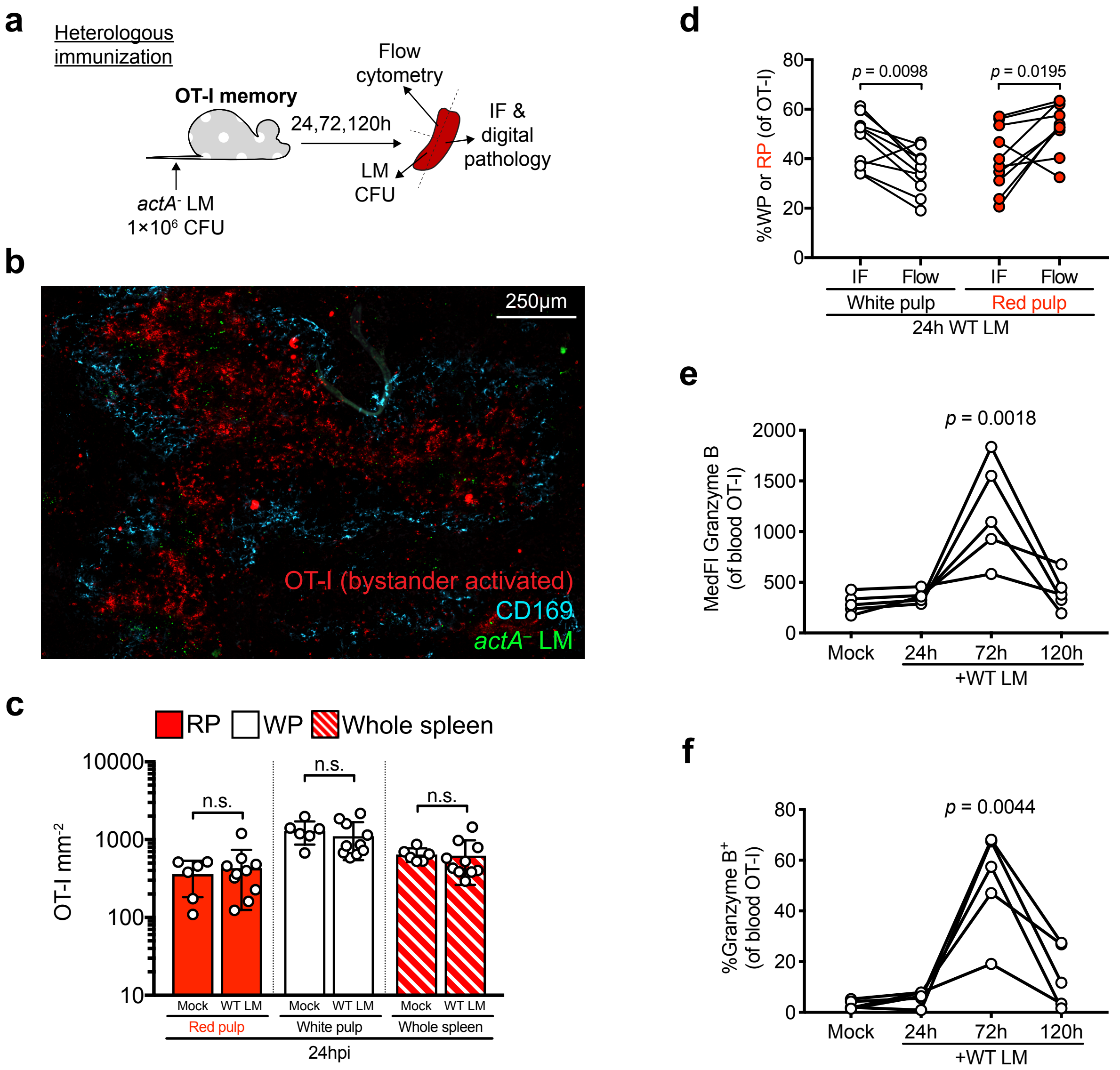

Supplementary Fig. 2. Memory CD8$^+$ T cells densely cluster at sites of early immune activation
(a) Schematic of heterologous (bystander activating) *actA*$^-$ LM immunization and subsequent tissue sampling. **(b)** Representative image of OT-I clustering and LM Ag within the white pulp (WP) of spleens from mice immunized with *actA*$^-$ LM 24 hours post infection. **(c)** OT-I densities within major splenic compartments calculated from IF and HALO enumeration from animals 24h post WT LM or mock immunization. **(d)** Frequency of splenic white pulp (WP) and red pulp (RP) OT-I T cells detected by i.v. staining and flow cytometric analysis versus IF paired with digital pathology analysis at 24h post WT LM infection. **(e-f)** Granzyme B **(e)** MedFI and **(f)** expression frequency in blood OT-I cells 24, 72, and 120h after WT LM immunization. **(b)** is representative of n=3 spleens. In **(c)** each symbol represents one splenic tissue compartment from one mouse from 4 experiments (n=10 WT LM immunized; n=6 unimmunized), summaries shown are mean ± SD; indicated are statistical significances by Mann-Whitney test with α = 0.05. In **(d)** each connected symbol pair represents one splenic compartment from one mouse from 3 experiments (n=10). Indicated are statistical significances by Wilcoxon matched-pairs signed rank test. In **(e** and **f)** each symbol represents 2-5 animals concatenated from the same technical replicate (replicate n=5). Symbols are connected by matching technical replicate. Indicated are statistical significances with adjusted *p* values below 0.05 by Dunn's multiple comparison test against mock-immunized events. Source data are provided as a Source Data file.

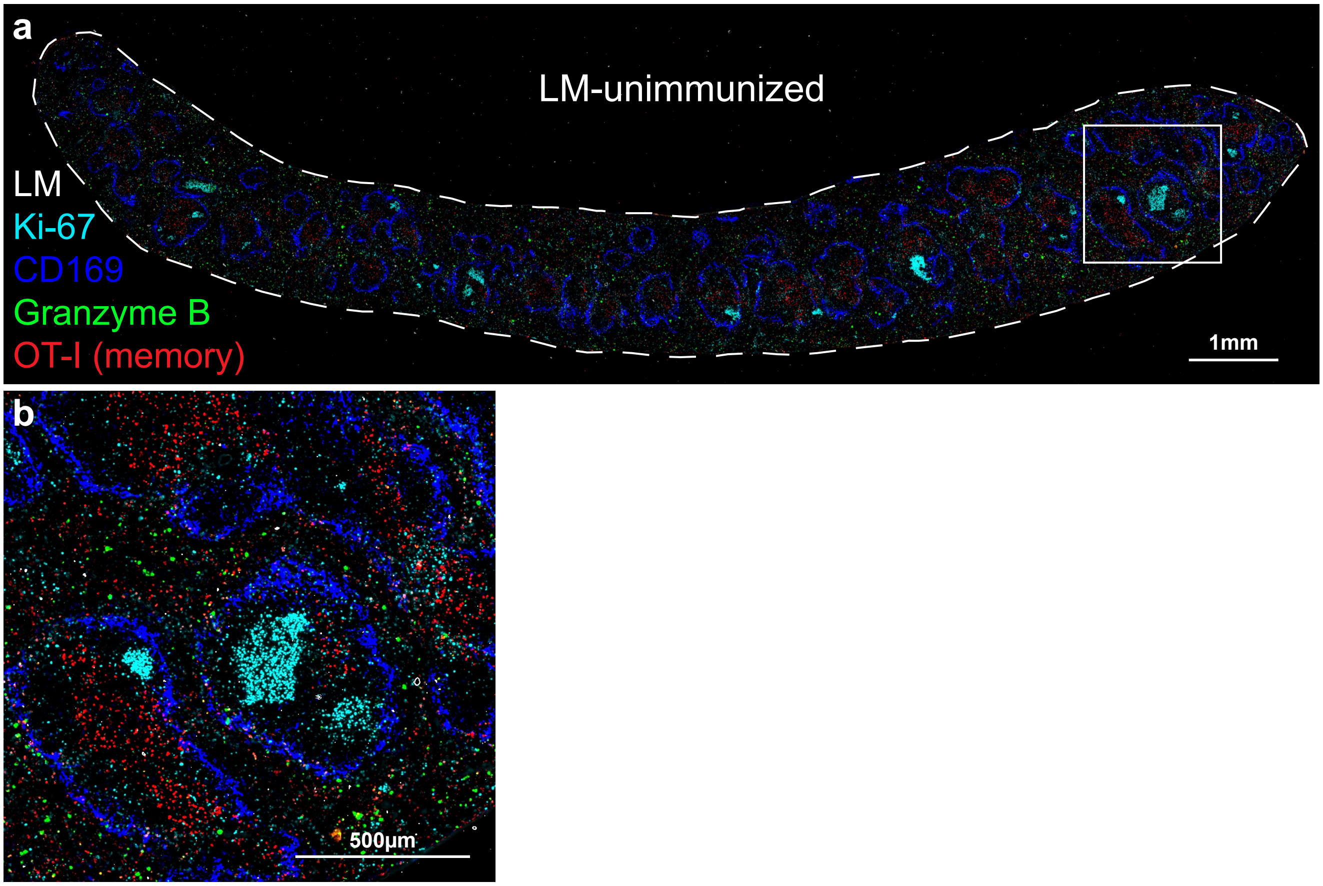

Supplementary Fig. 3. Clustering OT-I memory T cells do not exhibit signs of proliferation. (a, b) Representative image of LM-unimmunized spleen from serially sectioned 8µm slides stained for geographic markers: LM (white) and MMM (blue); and OT-I phenotyping: OT-I (red), granzyme B (green), and Ki-67 (cyan). In **(a, b)** image contrast was increased at an equal level as in Fig. 2a, b and tissue orientation was modified in Adobe Photoshop to overlay serially sectioned slides. All channels were merged and LM staining was outlined using “find edges” to increase visibility using ImageJ. Image is representative of (n=4).

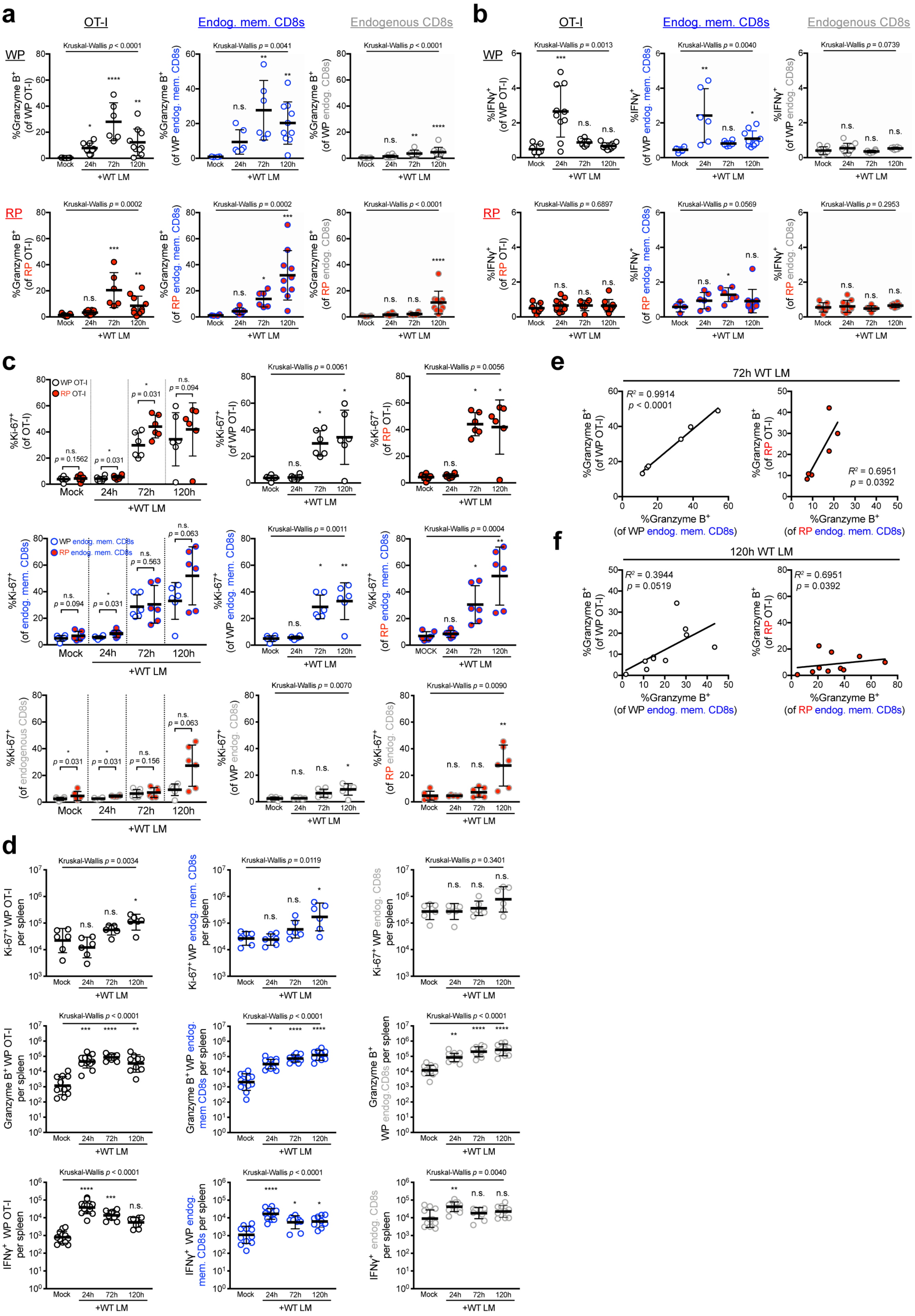
a
OT-I
Endog. mem. CD8s
Endogenous CD8s
WP
%Granzyme B+ (of WP OT-I)
%Granzyme B+ (of WP endog. mem. CD8s)
%Granzyme B+ (of WP endog. CD8s)
Kruskal-Wallis p < 0.0001
Kruskal-Wallis p = 0.0041
Kruskal-Wallis p < 0.0001
RP
%Granzyme B+ (of RP OT-I)
%Granzyme B+ (of RP endog. mem. CD8s)
%Granzyme B+ (of RP endog. CD8s)
Kruskal-Wallis p = 0.0002
Kruskal-Wallis p = 0.0002
Kruskal-Wallis p < 0.0001
b
OT-I
Endog. mem. CD8s
Endogenous CD8s
WP
%IFNγ+ (of WP OT-I)
%IFNγ+ (of WP endog. mem. CD8s)
%IFNγ+ (of WP endog. CD8s)
Kruskal-Wallis p = 0.0013
Kruskal-Wallis p = 0.0040
Kruskal-Wallis p = 0.0739
RP
%IFNγ+ (of RP OT-I)
%IFNγ+ (of RP endog. mem. CD8s)
%IFNγ+ (of RP endog. CD8s)
Kruskal-Wallis p = 0.6897
Kruskal-Wallis p = 0.0569
Kruskal-Wallis p = 0.2953
Mock
24h
72h
120h
+WT LM
n.s.
c
WP OT-I
RP OT-I
%Ki-67+ (of OT-I)
p = 0.1562
p = 0.031
p = 0.031
p = 0.094
%Ki-67+ (of WP OT-I)
Kruskal-Wallis p = 0.0061
%Ki-67+ (of RP OT-I)
Kruskal-Wallis p = 0.0056
WP endog. mem. CD8s
RP endog. mem. CD8s
%Ki-67+ (of endog. mem. CD8s)
p = 0.094
p = 0.031
p = 0.563
p = 0.063
%Ki-67+ (of WP endog. mem. CD8s)
Kruskal-Wallis p = 0.0011
%Ki-67+ (of RP endog. mem. CD8s)
Kruskal-Wallis p = 0.0004
MOCK
%Ki-67+ (of endogenous CD8s)
p = 0.031
p = 0.031
p = 0.156
p = 0.063
%Ki-67+ (of WP endog. CD8s)
Kruskal-Wallis p = 0.0070
%Ki-67+ (of RP endog. CD8s)
Kruskal-Wallis p = 0.0090
d
Ki-67+ WP OT-I per spleen
Kruskal-Wallis p = 0.0034
Ki-67+ WP endog. mem. CD8s per spleen
Kruskal-Wallis p = 0.0119
Ki-67+ WP endog. CD8s per spleen
Kruskal-Wallis p = 0.3401
Granzyme B+ WP OT-I per spleen
Kruskal-Wallis p < 0.0001
Granzyme B+ WP endog. mem CD8s per spleen
Kruskal-Wallis p < 0.0001
Granzyme B+ WP endog CD8s per spleen
Kruskal-Wallis p < 0.0001
IFNγ+ WP OT-I per spleen
Kruskal-Wallis p < 0.0001
IFNγ+ WP endog. mem. CD8s per spleen
Kruskal-Wallis p < 0.0001
IFNγ+ endog. CD8s per spleen
Kruskal-Wallis p = 0.0040
e
72h WT LM
R2 = 0.9914
p < 0.0001
%Granzyme B+ (of WP OT-I)
%Granzyme B+ (of WP endog. mem. CD8s)
R2 = 0.6951
p = 0.0392
%Granzyme B+ (of RP OT-I)
%Granzyme B+ (of RP endog. mem. CD8s)
f
120h WT LM
R2 = 0.3944
p = 0.0519
%Granzyme B+ (of WP OT-I)
%Granzyme B+ (of WP endog. mem. CD8s)
R2 = 0.6951
p = 0.0392
%Granzyme B+ (of RP OT-I)
%Granzyme B+ (of RP endog. mem. CD8s)

Supplementary Fig. 4. Site-specific bystander-activation is temporally unique and conserved in endogenous memory $CD8^+$ T cells. (a-c) Frequencies of **(a)** granzyme B^+, **(b)** $IFN\gamma^+$, or **(c)** Ki-67^+ OT-I T cells, endogenous memory $CD8^+$ T cells, and bulk (all) endogenous $CD8^+$ T cells within the white pulp (WP) or red pulp (RP) during heterologous WT LM immunization. **(d)** Counts of granzyme B^+, $IFN\gamma^+$, or Ki-67^+ OT-I T cells, endogenous memory $CD8^+$ T cells, and bulk endogenous $CD8^+$ T cells from WPs during heterologous WT LM immunization. **(e**, **f)** Scatter plots comparing magnitude of WP or RP OT-I and endogenous memory $CD8^+$ T cell responses at **(e)** 72 hours or **(f)** 120 hours after WT LM immunization. In **(a** and **b)** each symbol represents one mouse from 3 experiments (n=4 to 10 per condition per time point); summaries shown are mean ± SD and indicated are statistical significances by Kruskal-Wallis tests and Dunn's multiple comparisons test at * $p < 0.05$, ** $p < 0.01$, *** $p < 0.001$, **** $p < 0.0001$. In **(c)** each symbol represents one splenic compartment from one animal (left panels) or one animal (center and right panels) (n=6 from 2 technical replicates). Summaries shown are mean ± SD. In left panels, indicated are statistical significances by Wilcoxon matched-pairs signed rank test. For center and right panels, indicated are statistical significances by Kruskal-Wallis tests and Dunn's multiple comparisons test against LM-unimmunized animals (mock) at * $p < 0.05$, ** $p < 0.01$, *** $p < 0.001$, **** $p < 0.0001$. In **(d)** each symbol represents one animal (n=6 to 13 per time point, 2 to 5 technical replicates). Summaries shown are mean ± SD; indicated are statistical significances by Kruskal-Wallis tests and Dunn's multiple comparisons test at * $p < 0.05$, ** $p < 0.01$, *** $p < 0.001$, **** $p < 0.0001$. In **(e** and **f)** each symbol represents one mouse from 3 experiments (n=6-10). Indicated are statistical significances by linear regression. Source data are provided as a Source Data file.

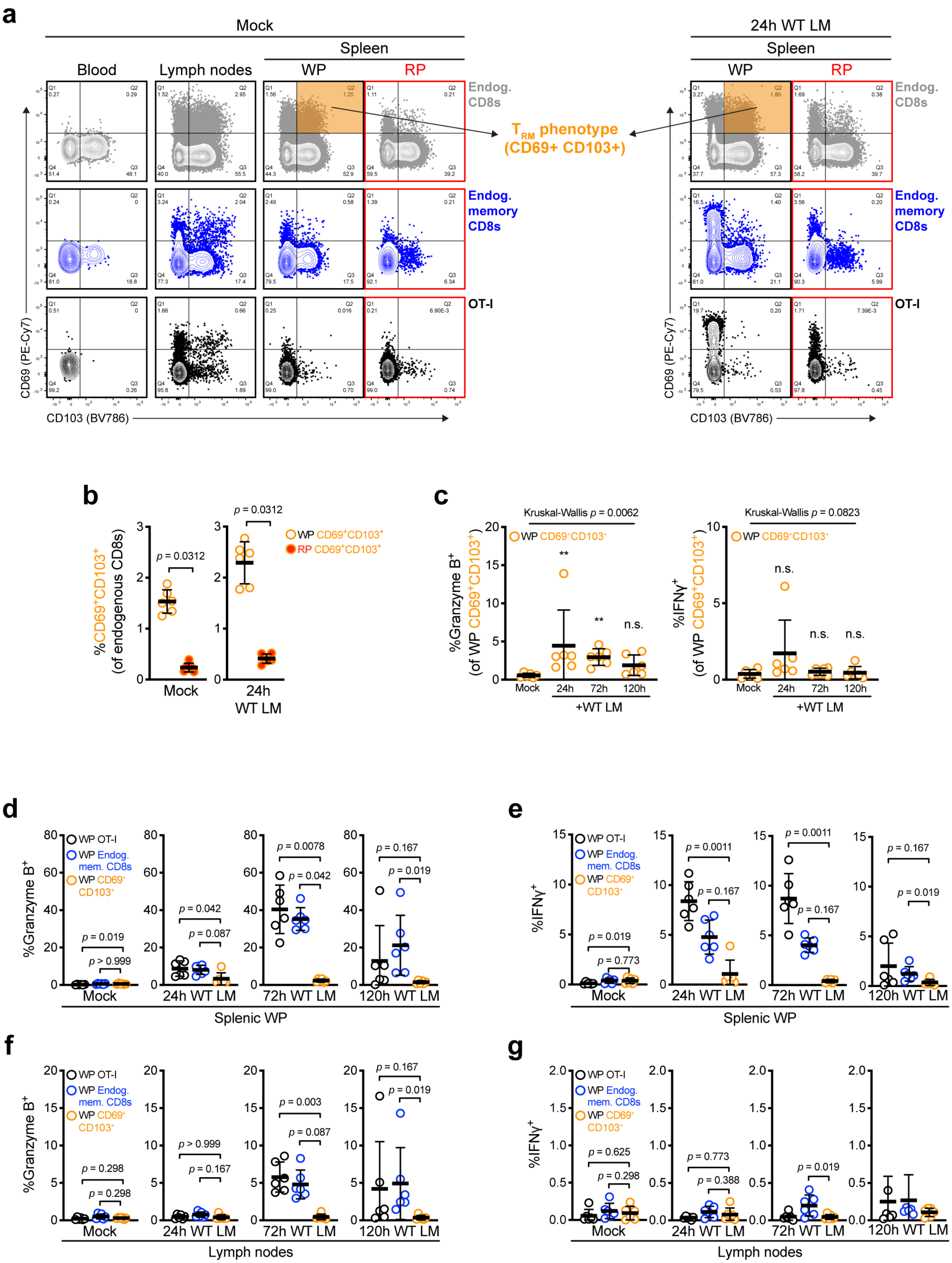
a
Mock
Spleen
Blood
Lymph nodes
WP
RP
Endog. CD8s
Endog. memory CD8s
OT-I
T_RM phenotype (CD69+ CD103+)
24h WT LM
CD69 (PE-Cy7)
CD103 (BV786)
b
%CD69+CD103+ (of endogenous CD8s)
p = 0.0312
WP CD69+CD103+
RP CD69+CD103+
Mock
24h WT LM
c
Kruskal-Wallis p = 0.0062
Kruskal-Wallis p = 0.0823
%Granzyme B+ (of WP CD69+CD103+)
%IFNγ+ (of WP CD69+CD103+)
**
n.s.
Mock
24h
72h
120h
+WT LM
d
e
f
g
WP OT-I
WP Endog. mem. CD8s
WP CD69+ CD103+
%Granzyme B+
%IFNγ+
p = 0.019
p > 0.999
p = 0.042
p = 0.087
p = 0.0078
p = 0.042
p = 0.167
p = 0.773
p = 0.0011
p = 0.298
p = 0.003
p = 0.625
p = 0.388
Mock
24h WT LM
72h WT LM
120h WT LM
Splenic WP
Lymph nodes

Supplementary Fig. 5. T_{RM}-phenotype CD69$^+$CD103$^+$ cells are less responsive to *in vivo* bystander activation. (a) Gating strategy demonstrating CD69 CD103 co-expression patterns in bulk endogenous CD8$^+$ T cells (grey), endogenous memory CD8$^+$ T cells (blue), or OT-I T cells (black) isolated from blood, lymph node (LN), splenic white pulp (WP), or circulatory splenic red pulp (RP, red outline). Left panel set depicts an LM-unimmunized animal and right panel set depicts an animal 24 hours after WT LM immunization; T_{RM} phenotype cells (i.e. CD69$^+$CD103$^+$) are highlighted in orange. **(b)** frequencies of WP (orange outline, white fill) and RP (orange outline, red fill) CD69$^+$CD103$^+$ endogenous CD8$^+$ cells. **(c)** Frequencies of granzyme B$^+$ (left) or IFNγ$^+$ (right) CD69$^+$CD103$^+$ endogenous CD8$^+$ T cells during WT LM immunization. **(d-g)** Expression of **(d**, **f)** granzyme B and **(r**, **g)** IFNγ in OT-I T cells (black outline), endogenous memory CD8$^+$ T cells (blue outline), and CD69$^+$CD103$^+$ endogenous CD8$^+$ T cells from **(d**, **e)** splenic WP or **(f**, **g)** LNs during WT LM immunization. In **(b-g)** summaries shown are mean ± SD; each symbol represents one cell population from one animal (n=6 from 2 technical replicates). In **(b)**, indicated statistical significances were determined by Wilcoxon matched-pairs signed rank test. In **(c)**, indicated statistical significances were determined by Kruskal-Wallis tests and Dunn's multiple comparisons test at * $p < 0.05$, ** $p < 0.01$, *** $p < 0.001$, **** $p < 0.0001$. In **(d-f)**, indicated statistical significances are adjusted *p* values determined by Dunn's multiple comparison tests. Source data are provided as a Source Data file.

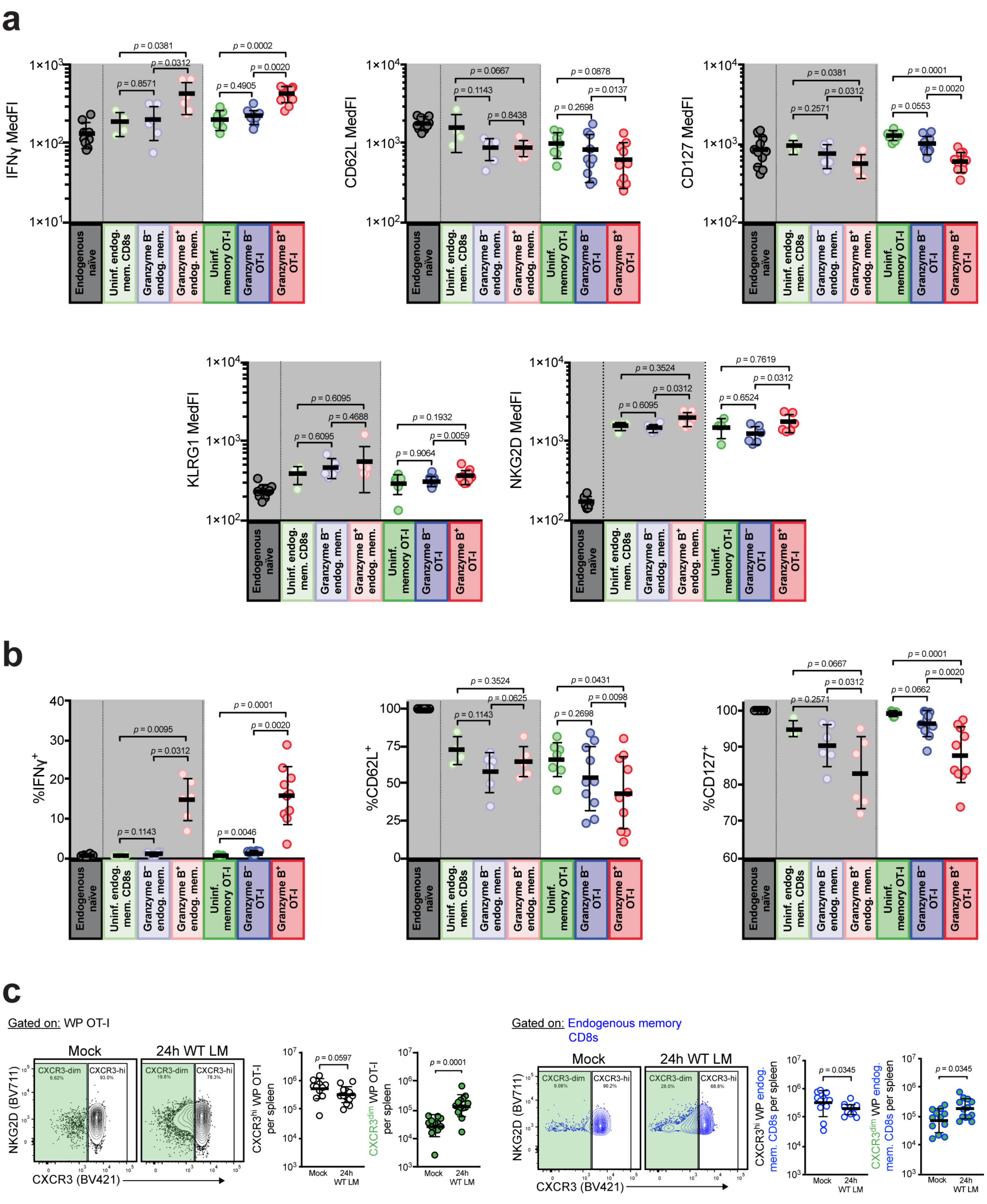
a
IFNγ MedFI
CD62L MedFI
CD127 MedFI
KLRG1 MedFI
NKG2D MedFI
Endogenous naïve
Uninf. endog. mem. CD8s
Granzyme B⁻ endog. mem.
Granzyme B⁺ endog. mem.
Uninf. memory OT-I
Granzyme B⁻ OT-I
Granzyme B⁺ OT-I
p = 0.0381
p = 0.0312
p = 0.8571
p = 0.0002
p = 0.0020
p = 0.4905
p = 0.0667
p = 0.1143
p = 0.8438
p = 0.0878
p = 0.0137
p = 0.2698
p = 0.0381
p = 0.0312
p = 0.2571
p = 0.0001
p = 0.0020
p = 0.0553
p = 0.6095
p = 0.4688
p = 0.6095
p = 0.1932
p = 0.0059
p = 0.9064
p = 0.3524
p = 0.0312
p = 0.6095
p = 0.7619
p = 0.0312
p = 0.6524
b
%IFNγ⁺
%CD62L⁺
%CD127⁺
p = 0.0095
p = 0.0312
p = 0.1143
p = 0.0001
p = 0.0020
p = 0.0046
p = 0.3524
p = 0.0625
p = 0.1143
p = 0.0431
p = 0.0098
p = 0.2698
p = 0.0667
p = 0.0312
p = 0.2571
p = 0.0001
p = 0.0020
p = 0.0662
c
Gated on: WP OT-I
Mock
24h WT LM
CXCR3-dim
CXCR3-hi
NKG2D (BV711)
CXCR3 (BV421)
CXCR3hi WP OT-I per spleen
CXCR3dim WP OT-I per spleen
p = 0.0597
p = 0.0001
Gated on: Endogenous memory CD8s
CXCR3hi WP endog. mem. CD8s per spleen
CXCR3dim WP endog. mem. CD8s per spleen
p = 0.0345
p = 0.0345

Supplementary Fig. 6. Bystander activated CD8$^+$ T cells are phenotypically distinct. (a) Median fluorescence intensity (MedFI) or **(b)** expression frequency for phenotypic and functional markers assessed by flow cytometric analysis in white pulp (WP) CD8$^+$ T cell populations (see Fig. 4a, b for population gating). Endogenous memory CD8$^+$ and memory OT-I T cells (light green and green, respectively) are from LM-unimmunized animals. Endogenous naïve (grey), granzyme B$^+$ (i.e. bystander activated) and granzyme B$^-$ (i.e. non-bystander activated) endogenous memory CD8$^+$ cells (light red and light blue, respectively) and OT-I cells (red and blue, respectively) are from animals 24 hours after WT LM immunization. Endogenous cell populations are shown in columns with grey background. **(a)** Identification and counts of CXCR3^{dim} (green fill) and CXCR3^{hi} (white fill) populations in WP OT-I (black outlines and contour plots) and WP endogenous memory CD8$^+$ T cells (blue outlines and contour plots). In figures **(a**, **b)** a single point represents a specific population from a single animal (n=6-10), summaries shown are mean ± SD. Indicated are statistical significances by Wilcoxon matched-pairs signed rank test. In figure **(C)** a single point represents a specific population from a single animal (n=12-13 per condition from 5 technical replicates). Summaries shown are mean ± SD; indicated are statistical significances by Mann-Whitney tests. Source data are provided as a Source Data file.

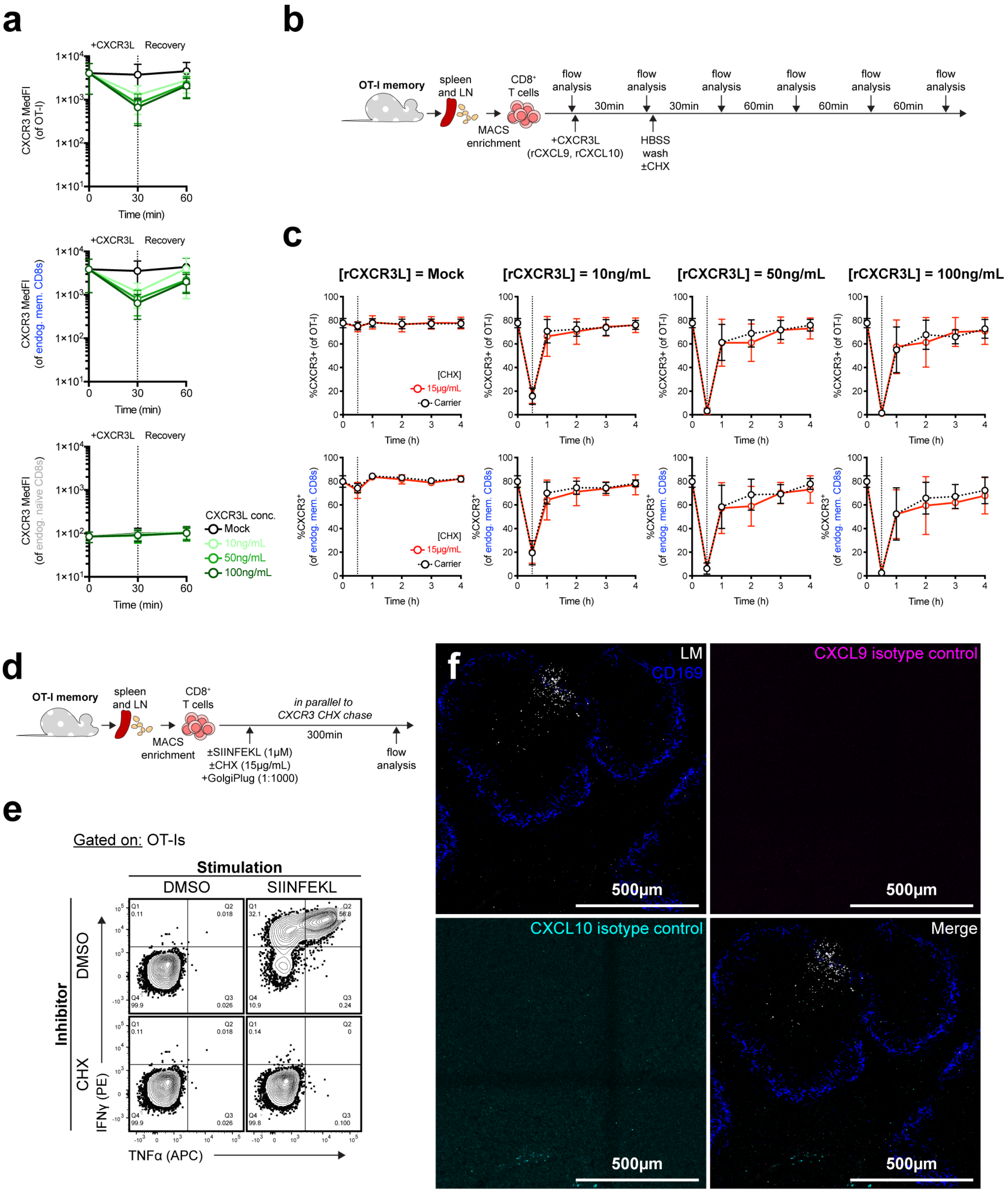

a
+CXCR3L
Recovery
CXCR3 MedFI (of OT-I)
CXCR3 MedFI (of endog. mem. CD8s)
CXCR3 MedFI (of endog. naive CD8s)
Time (min)
CXCR3L conc.
Mock
10ng/mL
50ng/mL
100ng/mL
b
OT-I memory
spleen and LN
MACS enrichment
CD8+ T cells
flow analysis
+CXCR3L (rCXCL9, rCXCL10)
HBSS wash ±CHX
30min
30min
60min
60min
60min
c
[rCXCR3L] = Mock
[rCXCR3L] = 10ng/mL
[rCXCR3L] = 50ng/mL
[rCXCR3L] = 100ng/mL
%CXCR3+ (of OT-I)
%CXCR3+ (of endog. mem. CD8s)
Time (h)
[CHX]
15μg/mL
Carrier
d
OT-I memory
spleen and LN
MACS enrichment
CD8+ T cells
in parallel to CXCR3 CHX chase
300min
±SIINFEKL (1μM)
±CHX (15μg/mL)
+GolgiPlug (1:1000)
flow analysis
e
Gated on: OT-Is
Stimulation
DMSO
SIINFEKL
Inhibitor
DMSO
CHX
IFNγ (PE)
TNFα (APC)
f
LM
CD169
CXCL9 isotype control
CXCL10 isotype control
Merge
500μm

Supplementary Fig. 7. Murine CXCR3 is recycled after ligand-mediated internalization in the absence of protein synthesis. (a) Median fluorescence intensity (MedFI) values for CXCR3 in OT-I T cells (top), endogenous memory $CD8^+$ T cells (middle), and endogenous naïve $CD8^+$ T cells (bottom) prior to rCXCR3L (rCXCL9 and rCXCL10) stimulation (0min), after rCXCR3L stimulation (30min), and 30 minutes after rCXCR3L removal (60min) (see Fig. 5a for experiment diagram). Symbols and line connectors are color-coded by rCXCR3L concentration (mock, black; 10ng/mL each, light green; 50ng/mL each, medium green; 100ng/mL each, dark green). **(b)** Schematic illustrating cycloheximide (CHX) chase to test if mouse CXCR3 is restored through *de novo* protein synthesis. **(c)** CXCR3 frequency in OT-I (top row) or endogenous memory $CD8^+$ T cells (bottom row) during CHX (red, solid line) or carrier (black, dashed line) treatment. Vertical reference line signifies end of rCXCR3L stimulation and addition of CHX-containing media. **(d)** Schematic illustrating peptide stimulation assay to control for CHX performance. **(e)** Representative flow plot depicting OT-I production of effector cytokines via cognate Ag (SIINFEKL peptide) in the presence or absence of CHX. **(f)** Representative image depicting CXCL9 and CXCL10 IF isotype control staining in same foci from serial sections in Fig 5e, f. In **(a** and **c)** each symbol represents the mean from two replicates, ± SD, with lines connecting means. **(f)** is representative of n=3 and image processing was identical to Fig 5e, f: Image orientation was modified in Adobe Photoshop to permit overlaying of serially-sectioned slides; contrast was equally increased amongst all individual images; CXCL9 and CXCL10 isotype control images were outlined using ImageJ command “find edges” prior to merging. Source data are provided as a Source Data file.

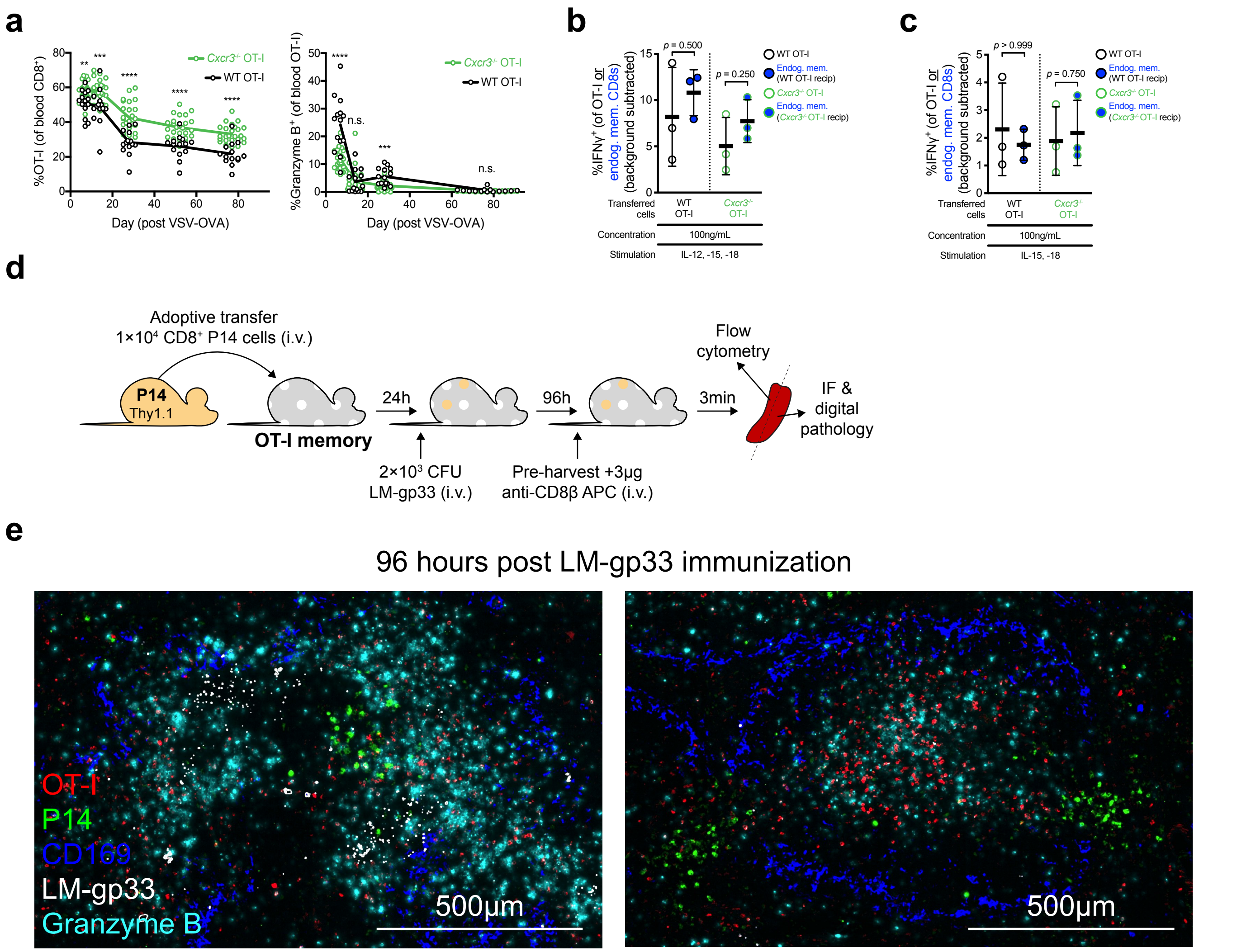

Supplementary Fig. 8. Bystander and *de novo* Ag-specific responses temporospatially overlap. **(a)** OT-I T cell frequency and granzyme B$^+$ expression in circulating WT (black) and *Cxcr3*$^{-/-}$ (green) OT-I T cells following primary VSV-OVA infection used to generate memory OT-I T cells. **(b-c)** Background-subtracted IFNγ expression levels in OT-I and endogenous memory CD8$^+$ T cells 6 hours after stimulation with **(b)** 100ng/mL each of IL-12, -15, -18 or **(c)** 100ng/mL each of IL-15, -18. **(d)** Schematic of adoptive transfer of naïve P14 TCR transgenic cells and subsequent LM-gp33 immunization (provides cognate Ag for P14 cells, but bystander activates OT-I cells) and subsequent tissue sampling. **(e)** IF of splenic WP 96 hours after LM-gp33 immunization, with P14 cells shown in green, OT-I cells in red, granzyme B expression in cyan, CD169 in blue, and LM-gp33 in white. In figure **(a)** each symbol is representative of one animal, summaries shown are lines connecting means at each time point. n=14 WT OT-I recipients; n=25 *Cxcr3*$^{-/-}$ OT-I recipients. Indicated are statistical significances at *p < 0.05, **p < 0.01, *** < 0.001 comparing WT and *Cxcr3*$^{-/-}$ OT-I cells at the same time point by Mann-Whitney tests. In **(b** and **c)** each symbol is representative of a cell subset from a single animal (n=3, three technical replicates); summaries shown are mean ± SD. Indicated statistical significances were calculated by Wilcoxon matched-pairs signed rank test. In **(e)** separate images are from different mice and representative of n=3 animals. Image contrast was increased at an equal level and tissue orientation was modified in Adobe Photoshop to overlay serially-sectioned slides. All channels were merged and LM staining was outlined using "find edges" to increase visibility using ImageJ. Source data are provided as a Source Data file.

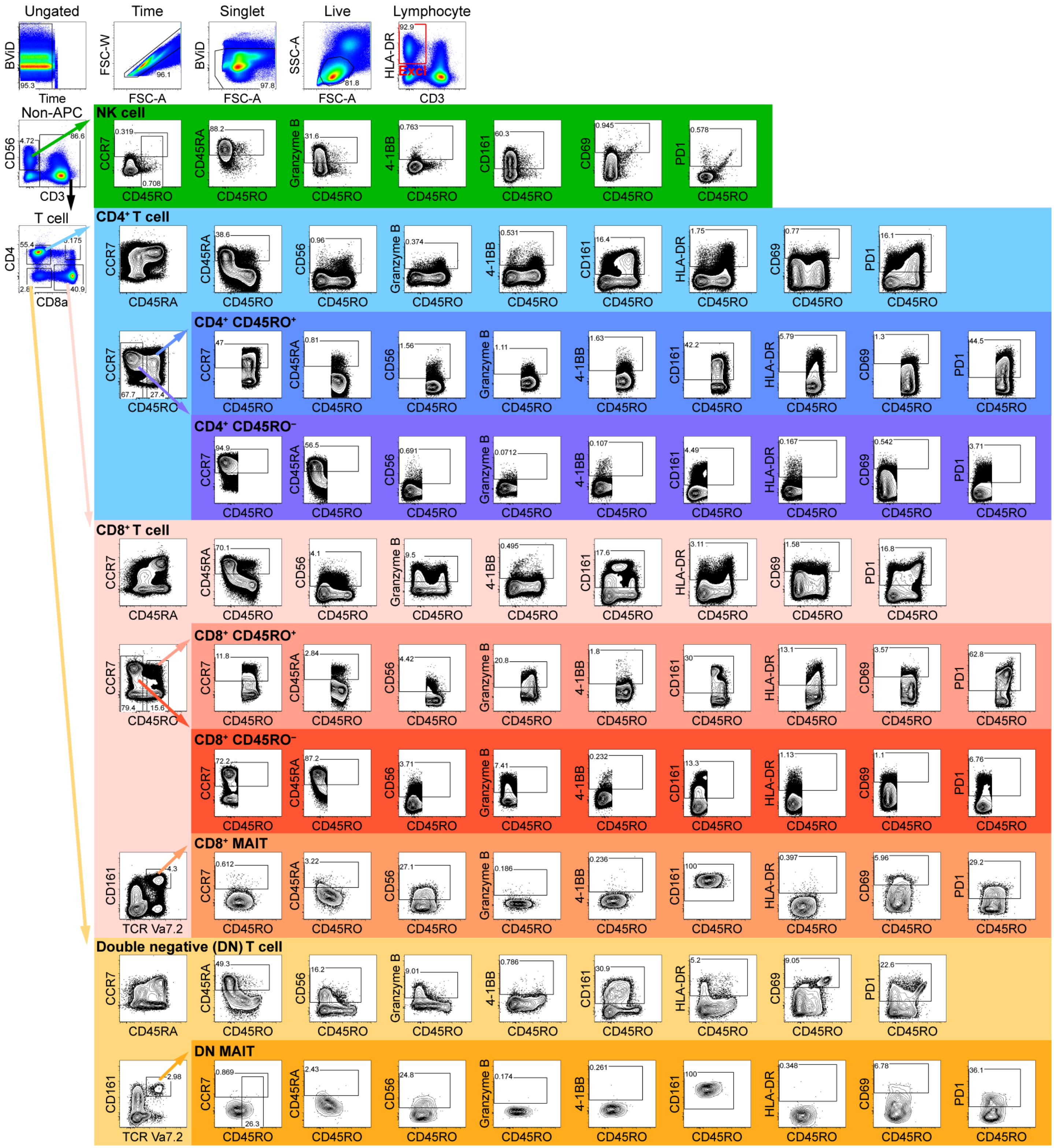

Supplementary Fig. 9. Flow cytometry gating strategy for HVTN 205/908 data. Gating strategy used for flow cytometry data in Supplementary Fig. 1.

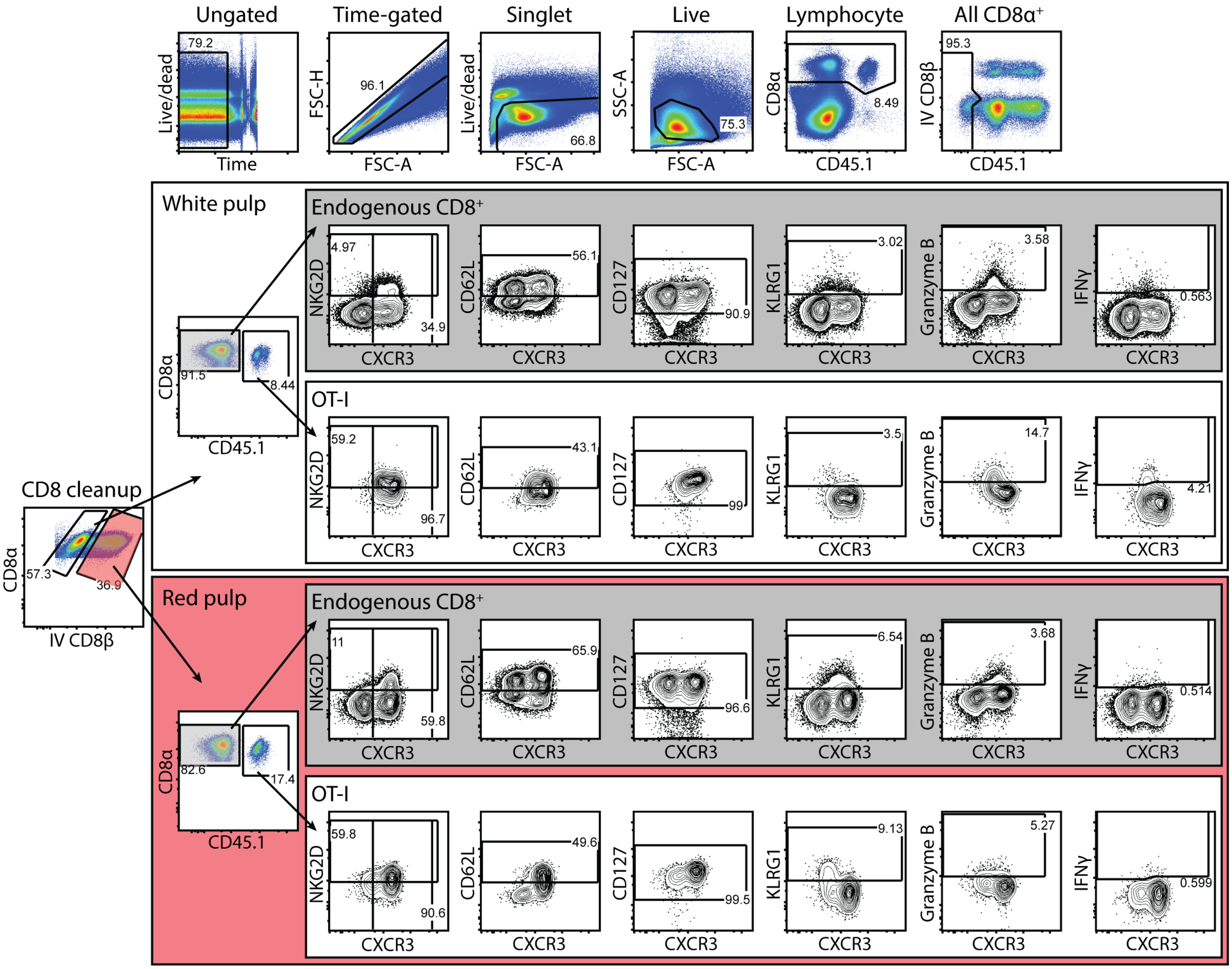

Supplementary Fig. 10. Flow cytometry gating strategy for identifying and phenotyping bystander activated OT-I and endogenous CD8^{+} T cells. Gating strategy used for flow cytometry data in Fig. 3, 4, 6, and Supplementary Fig. 2, 4, 5, 6, 8.

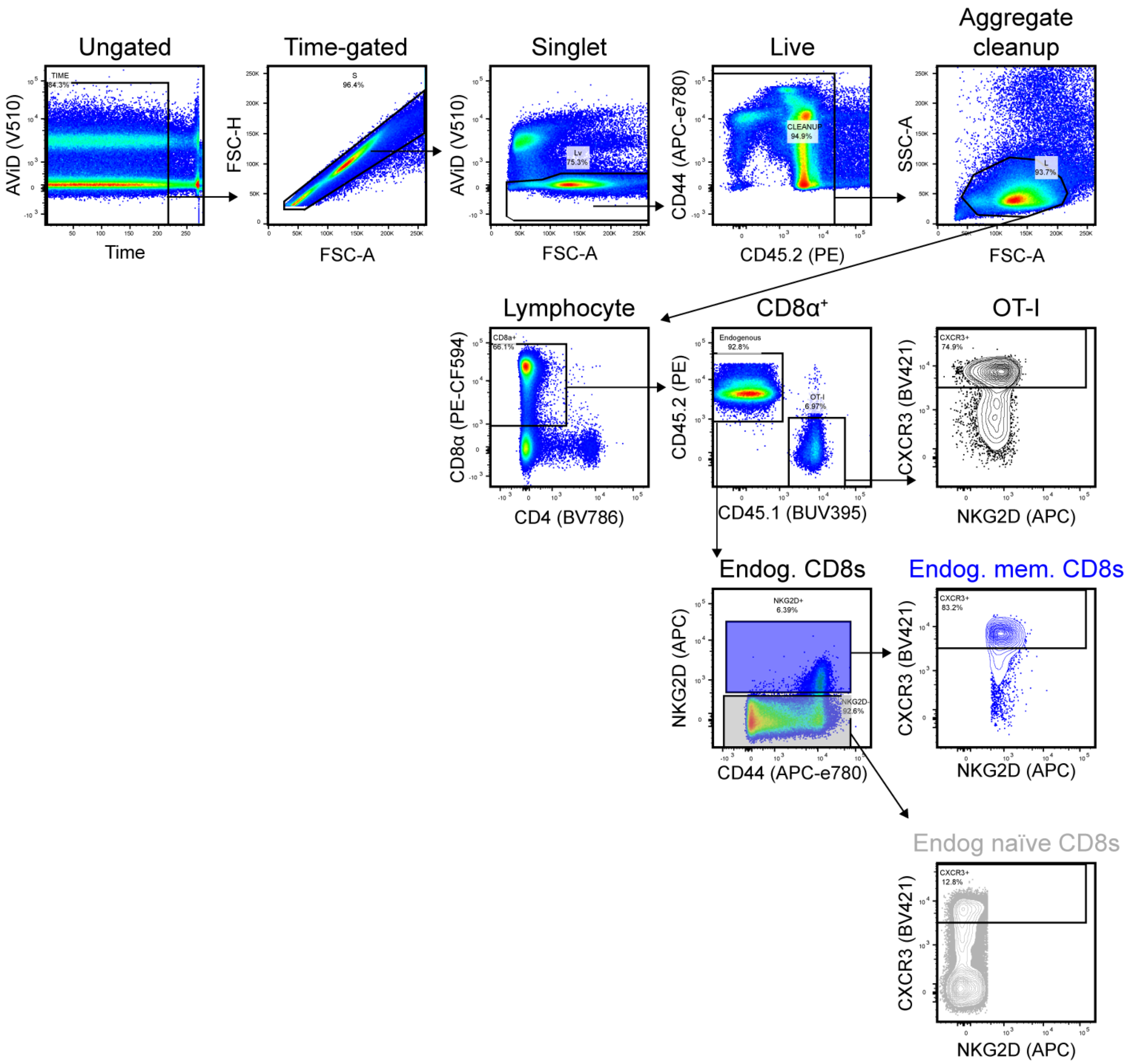

Supplementary Fig. 11. Flow cytometry gating strategy for *in vitro* stimulations with recombinant CXCR3 ligands. Gating strategy used for flow cytometry data in Fig. 5 and Supplementary Fig. 7.

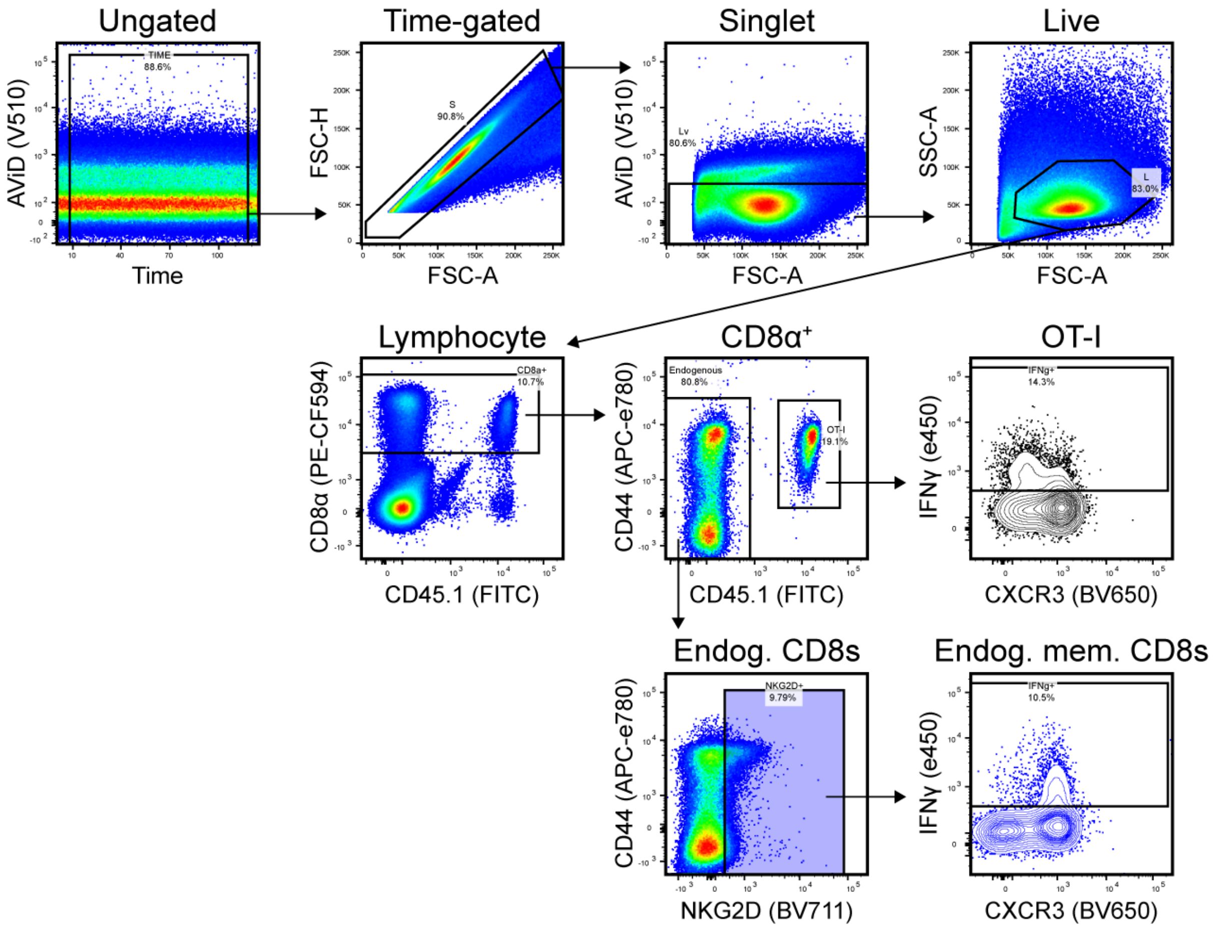

Supplementary Fig. 12. Flow cytometry gating strategy for *in vitro* stimulations with bystander-activating cytokines. Gating strategy used for flow cytometry data in Fig. 6 and Supplementary Fig. 8.

**Human bystander activation flow cytometry panel**				
**Antigen**	**Conjugate**	**Clone**	**Vendor**	**Dilution**
Viability stain (in 1x PBS)				
LIVE/DEAD blue fixable viability dye (BViD)	U450	N/A	Thermo Fisher Scientific	1:500
Tetramer stain (in FACSWash)				
GILGFVFTL (IAV $M1_{58-66}$) HLA-A*02 tetramer	PE	N/A	Fred Hutch	1:200
APC YVLDHLIVV (EBV $BRLF1_{190-198)}$ HLA-A*02 tetramer	APC	N/A	Fred Hutch	1:200
Human TruStain FcX	N/A	N/A	BioLegend	1:20
Surface stain (in FACSWash)				
CD8	BUV395	RPA-T8	BD Biosciences	1:25
HLA-DR	BUV661	G46-6	BD Biosciences	1:50
CD69	BUV737	FN50	BD Biosciences	1:100
4-1BB (CD137)	BV421	4B4-1	BD Biosciences	1:25
CD45RA	BV510	HI100	BD Biosciences	1:80
CD161	BV605	HP-G310	BioLegend	3:50
CD4	BV711	OKT4	BioLegend	1:80
CD3	BV786	SK7	BD Biosciences	1:50
TCR Vα7.2	FITC	3C10	BioLegend	1:10
CD56	BB700	B159	BD Biosciences	1:50
CCR7 (CD197)	PE-CF594	150503	BD Biosciences	1:20
PD-1 (CD279)	PE-Cy7	EH12.1	BD Biosciences	1:50
CD45RO	APC-H7	UCHL1	BD Biosciences	1:25
Human TruStain FcX	N/A	N/A	BioLegend	1:20
BD Cytofix/Cytoperm fixation				
Intracellular stain (in 1x BD Perm/Wash)				
Granzyme B	Alexa Fluor 700	GB11	BD Biosciences	1:80
Human TruStain FcX	N/A	N/A	BioLegend	1:20

Supplementary Table 1. Flow cytometry panel for HVTN 205/908 samples. Panel used for flow cytometry data in Supplementary Fig. 1, 9. Tetramer data not shown due to limited number of HLA-A*02 PBMC donors and tetramer-specific cells.

Murine general bystander activation flow cytometry panel				
**Antigen**	**Conjugate**	**Clone**	**Vendor**	**Dilution**
i.v. stain				
CD8β	APC	eBioH35-17.2	Thermo Fisher Scientific	3μg/mouse
Viability stain (in 1x PBS)				
LIVE/DEAD aqua fixable viability dye (AViD)	V510	N/A	Thermo Fisher Scientific	1:500
Surface stain (in FACSWash+)				
CD45.1	BUV395	A20	BD Biosciences	1:200
CXCR3	BV421	CXCR3-173	BD Biosciences	1:100
KLRG1	BV605	2F1	BD Biosciences	1:200
NKG2D	BV711	CX5	BD Biosciences	1:200
CD62L	FITC	MEL-14	Thermo Fisher Scientific	1:300
CD8α	PE-CF594	53-6.7	BD Biosciences	1:300
CD127	APC-e780	A7R34	Thermo Fisher Scientific	1:100
CD16/32 (Fc block)	Purified	24.G2	BD Biosciences	1:300
BD Cytofix/Cytoperm fixation				
Intracellular stain (in 1x BD Perm/Wash)				
IFNγ	BUV737	XMG1.2	BD Biosciences	1:100
Granzyme B	PE	GB11	Thermo Fisher Scientific	1:100
Ki-67[a]	PE-Cy7	16A8	BioLegend	1:200
Granzyme A[a]	PE-Cy7	GzA-3G8.5	Thermo Fisher Scientific	1:100
CD16/32 (Fc block)	Purified	24.G2	BD Biosciences	1:300

[a]anti-Ki-67 or –Granzyme A were used alone (i.e. not in combination) in staining panel

Murine T_{RM} phenotyping bystander activation flow cytometry panel				
**Antigen**	**Conjugate**	**Clone**	**Vendor**	**Dilution**
i.v. stain				
CD8β	APC	eBioH35-17.2	Thermo Fisher Scientific	3μg/mouse
Viability stain (in 1x PBS)				
LIVE/DEAD aqua fixable viability dye (AViD)	V510	N/A	Thermo Fisher Scientific	1:500
Surface stain (in FACSWash+)				
CD45.1	BUV395	A20	BD Biosciences	1:200
CXCR3	BV421	CXCR3-173	BD Biosciences	1:100
PD-1	BV605	29F.1A12	BioLegend	1:150
NKG2D	BV711	CX5	BD Biosciences	1:200
CD103	BV786	M290	BD Biosciences	1:100
CD8α	PE-CF594	53-6.7	BD Biosciences	1:300
CD69	PE-Cy7	H1.2F3	Thermo Fisher Scientific	1:100
CD45.2	Alexa Fluor 700	104	Thermo Fisher Scientific	1:300
CD16/32 (Fc block)	Purified	24.G2	BD Biosciences	1:300
Fixation in 1x eBioscience FOXP3 fixation/permeabilization buffer				
Intracellular stain (in 1x FOXP3 permeabilization buffer)				
IFNγ	BUV737	XMG1.2	BD Biosciences	1:100
Ki-67	Alexa Fluor 488	11F6	BioLegend	1:200
Granzyme B	PE	GB11	Thermo Fisher Scientific	1:100
CD16/32 (Fc block)	Purified	24.G2	BD Biosciences	1:300

Supplementary Table 2. Flow cytometry panels for identification and phenotyping of bystander activated OT-I and endogenous $CD8^+$ T cells. Top panel used for data in Fig. 3, 4, 6, and Supplementary Fig. 2, 4, 6, 8, 10. Bottom panel used for data in Supplementary Fig. 4, 5.

Murine CXCR3L stimulation flow cytometry panel				
**Antigen**	**Conjugate**	**Clone**	**Vendor**	**Dilution**
Viability stain (in 1x PBS)				
LIVE/DEAD aqua fixable viability dye (AViD)	V510	N/A	Thermo Fisher Scientific	1:500
Surface stain (in FACSWash+)				
CD45.1	BUV395	A20	BD Biosciences	1:200
CXCR3	BV421	CXCR3-173	BD Biosciences	1:100
CD44	FITC	IM7	Thermo Fisher Scientific	1:300
CD45.2	PE	104	Thermo Fisher Scientific	1:300
CD8α	PE-CF594	53-6.7	BD Biosciences	1:300
NKG2D	APC	CX5	Thermo Fisher Scientific	1:100
CD16/32 (Fc block)	Purified	24.G2	BD Biosciences	1:300
BD Cytofix/Cytoperm fixation				

Murine CHX intracellular cytokine staining flow cytometry panel				
**Antigen**	**Conjugate**	**Clone**	**Vendor**	**Dilution**
Viability stain (in 1x PBS)				
LIVE/DEAD aqua fixable viability dye (AViD)	V510	N/A	Thermo Fisher Scientific	1:500
Surface stain (in FACSWash+)				
CD45.1	BUV395	A20	BD Biosciences	1:200
CXCR3	BV421	CXCR3-173	BD Biosciences	1:100
CD4	BV786	GK1.5	BD Biosciences	1:200
CD44	FITC	IM7	Thermo Fisher Scientific	1:300
CD8α	PE-CF594	53-6.7	BD Biosciences	1:300
CD45.2	Alexa Fluor 700	104	Thermo Fisher Scientific	1:300
CD16/32 (Fc block)	Purified	24.G2	BD Biosciences	1:300
Fixation in 1x eBioscience FOXP3 fixation/permeabilization buffer				
Intracellular stain (in 1x FOXP3 permeabilization buffer)				
IFNγ	PE	XMG1.2	BD Biosciences	1:100
TNFα	Alexa Fluor 647	MP6-XT22	BD Biosciences	1:100
CD16/32 (Fc block)	Purified	24.G2	BD Biosciences	1:300

Supplementary Table 3. Flow cytometry panels for CXCR3L stimulation and CHX chase assays. Top panel used for data in Fig. 5 and Supplementary Fig. 7, 11. Lower panel used for data in Supplementary Fig. 7.

**Murine functional panel for bystander-activating cytokine stimulations**				
**Antigen**	**Conjugate**	**Clone**	**Vendor**	**Dilution**
i.v. stain				
CD8β	APC	eBioH35-17.2	Thermo Fisher Scientific	3μg/mouse
Viability stain (in 1x PBS)				
LIVE/DEAD aqua fixable viability dye (AViD)	V510	N/A	Thermo Fisher Scientific	1:500
Surface stain (in FACSWash+)				
CXCR3	BV650	CXCR3-173	BioLegend	1:100
NKG2D	BV711	CX5	BD Biosciences	1:200
CD45.1	FITC	A20	Thermo Fisher Scientific	1:200
CXCR3	BV421	CXCR3-173	BD Biosciences	1:100
CD8α	PE-CF594	53-6.7	BD Biosciences	1:300
CD69	PE-Cy7	H1.2F3	Thermo Fisher Scientific	1:100
CD44	APC-e780	IM7	Thermo Fisher Scientific	1:300
CD16/32 (Fc block)	Purified	24.G2	BD Biosciences	1:300
Fixation in 1x eBioscience FOXP3 fixation/permeabilization buffer				
Intracellular stain (in 1x FOXP3 permeabilization buffer)				
IFNγ	eFluor 450	XMG1.2	Thermo Fisher Scientific	1:100
TNFα	Alexa Fluor 647	MP6-XT22	BD Biosciences	1:100
CD16/32 (Fc block)	Purified	24.G2	BD Biosciences	1:300

Supplementary Table 4. Flow cytometry panel for bystander-activating cytokine stimulations. Panel used for Fig 6 and Supplementary Fig. 8, 12.

**Murine bystander location IF panel**				
Tissue type: unfixed				
Post-sectioning fixation: -20°C acetone (5 minutes)				
Staining buffer: TBS + 5% human serum, 5% mouse serum				
Antigen	Conjugate	Clone	Vendor	Dilution
1° stain				
Mouse anti-mouse CD45.1	Biotin	A20	Thermo Fisher Scientific	1:50 (10μg/mL)
Rat anti-mouse CD169 (Siglec-1)	Alexa Fluor 594	3D6.112	BioLegend	1:200 (2.5μg/mL)
2° stain				
Streptavidin	Alexa Fluor 647	N/A	Thermo Fisher Scientific	1:100 (20μg/mL)
Rabbit anti-Listeria O Antiserum	N/A	N/A Polyclonal (Cat: 223021)	BD Biosciences	1:1,000-10,000[a]
3° stain				
Donkey anti-rabbit IgG	DyLight 488	Poly4064	BioLegend	1:100 (5μg/mL)
Nuclear counterstain				
DNA	DAPI	N/A	Thermo Fisher Scientific	1ng/mL

[a]Dilution range dependent on reagent lot

Supplementary Table 5. IF panel for detecting bystander OT-I T cells, CD169$^+$ MMM, and LM. Panel used for data in Fig. 2, 6, and Supplementary Fig. 2.

Murine bystander activation IF panel				
Tissue type: unfixed				
Post-sectioning fixation: 1:4 BD Cytofix in 1x PBS (5 minutes)				
Staining buffer: 1x BD Perm/Wash in TBS + 5% human serum, 5% mouse serum				
Antigen	Conjugate	Clone	Vendor	Dilution
1° stain				
Mouse anti-mouse CD45.1	Biotin	A20	Thermo Fisher Scientific	1:50 (10μg/mL)
Rat anti-human/mouse Ki-67	N/A	11F6	BioLegend	1:200 (2.5μg/mL)
Goat anti-mouse Granzyme B	N/A	N/A Polyclonal (Cat: AF1865)	R&D Systems	1:100 (2μg/mL)
2° stain				
Streptavidin	Alexa Fluor 594	N/A	BioLegend	1:100 (5μg/mL)
Chicken anti-rat IgG	Alexa Fluor 647	N/A Polyclonal (Cat: A21472)	Thermo Fisher Scientific	1:100 (20μg/mL)
Donkey anti-goat IgG	Alexa Fluor 488	N/A Polyclonal (Cat: A11055)	Thermo Fisher Scientific	1:100 (20μg/mL)
Nuclear counterstain				
DNA	DAPI	N/A	Thermo Fisher Scientific	1ng/mL

Supplementary Table 6. IF panel for detecting granzyme B and Ki-67 expression in OT-I T cells. Panel used for data in Fig. 3, 6, and Supplementary Fig. 3.

**Murine splenic architecture IF panel**				
Tissue type: fixed (1:4 BD Cytofix in 1x PBS; overnight)				
Post-sectioning fixation: N/A				
Staining buffer: TBS + 0.3% Triton X + 5% human serum, 5% mouse serum				
Antigen	Conjugate	Clone	Vendor	Dilution
1° stain				
Rat IgG2b anti-mouse CD11b	N/A	M1/70	BioLegend	1:100 (5μg/mL)
Rat IgG1 anti-mouse CD169 (Siglec-1)	Alexa Fluor 594	3D6.112	BioLegend	1:200 (2.5μg/mL)
Rabbit anti-Listeria O Antiserum	N/A	N/A Polyclonal (Cat: 223021)	BD Biosciences	1:1,000-10,000[a]
2° stain				
Mouse anti-rat IgG2b	Alexa Fluor 647	MRG2b-85	BioLegend	1:100 (5μg/mL)
Donkey anti-rabbit IgG	DyLight 488	Poly4064	BioLegend	1:100 (5μg/mL)
Nuclear counterstain				
DNA	DAPI	N/A	Thermo Fisher Scientific	1ng/mL

[a]Dilution range dependent on reagent lot

Supplementary Table 7. IF panel for detecting CD11b$^+$ cells, CD169$^+$ MMM, and LM. Panel used for data in Fig. 3, 5, and Supplementary Fig. 7.

**Murine CXCR3L IF panel**				
Tissue type: fixed (1:4 BD Cytofix in 1x PBS; overnight)				
Post-sectioning fixation: N/A				
Staining buffer: TBS + 0.3% Triton X + 5% human serum, 5% mouse serum				
Antigen	Conjugate	Clone	Vendor	Dilution
1° stain				
Armenian hamster anti-mouse CXCL9	eFluor 660	MIG-2F5.5	Thermo Fisher Scientific	1:50 (4μg/mL)
Goat anti-mouse CXCL10	N/A	N/A Polyclonal (Cat: AF-466-NA)	R&D Systems	1:50 (4μg/mL)
2° stain				
Chicken anti-goat IgG	Alexa Fluor 488	N/A Polyclonal (Cat: A21467)	Thermo Fisher Scientific	1:100 (20μg/mL)
Nuclear counterstain				
DNA	DAPI	N/A	Thermo Fisher Scientific	1ng/mL

Supplementary Table 8. IF panel for detecting CXCR3L. Panel used for data in Fig. 5.

Murine CXCR3L isotype control IF panel				
Tissue type: fixed (1:4 BD Cytofix in 1x PBS; overnight)				
Post-sectioning fixation: N/A				
Staining buffer: TBS + 0.3% Triton X + 5% human serum, 5% mouse serum				
Antigen	Conjugate	Clone	Vendor	Dilution
1° stain				
Armenian hamster IgG control	eFluor 660	eBio299Arm	Thermo Fisher Scientific	1:50 (4μg/mL)
Goat IgG isotype control	N/A	N/A Polyclonal (Cat: 02-6202)	Thermo Fisher Scientific	1:1250 (4μg/mL)
2° stain				
Chicken anti-goat IgG	Alexa Fluor 488	N/A Polyclonal (Cat: A21467)	Thermo Fisher Scientific	1:100 (20μg/mL)
Nuclear counterstain				
DNA	DAPI	N/A	Thermo Fisher Scientific	1ng/mL

Supplementary Table 9. IF panel for CXCR3L isotype controls. Panel used for data in Supplementary Fig. 7.

**Murine IFNγ bystander activation IF panel**				
Tissue type: fixed (1:4 BD Cytofix in 1x PBS; overnight)				
Post-sectioning fixation: N/A				
Staining buffer: TBS + 0.3% Triton X + 5% human serum, 5% mouse serum				
Antigen	Conjugate	Clone	Vendor	Dilution
1° stain				
Mouse anti-mouse CD45.1	Biotin	A20	Thermo Fisher Scientific	1:50 (10μg/mL)
Goat anti-mouse Granzyme B	N/A	N/A Polyclonal (Cat: AF1865)	R&D Systems	1:100 (2μg/mL)
Rat anti-mouse IFNγ	N/A	XMG1.2	Thermo Fisher Scientific	1:50 (4μg/mL)
2° stain				
Streptavidin	Alexa Fluor 647	N/A	Thermo Fisher Scientific	1:100 (20μg/mL)
Chicken anti-goat IgG	Alexa Fluor 488	N/A Polyclonal (Cat: A21467)	Thermo Fisher Scientific	1:100 (20μg/mL)
Chicken anti-rat IgG	Alexa Fluor 594	N/A Polyclonal (Cat: A21471)	Thermo Fisher Scientific	1:100 (20μg/mL)
Nuclear counterstain				
DNA	DAPI	N/A	Thermo Fisher Scientific	1ng/mL

Supplementary Table 10. IF panel for detecting IFNγ expression in bystander activated OT-I T cells. Panel used for data in Fig. 3.

Murine NKG2D bystander activation IF panel				
Tissue type: fixed (1:4 BD Cytofix in 1x PBS; overnight)				
Post-sectioning fixation: N/A				
Staining buffer: TBS + 0.3% Triton X + 5% human serum, 5% mouse serum				
Antigen	Conjugate	Clone	Vendor	Dilution
1° stain				
Mouse anti-mouse CD45.1	Alexa Fluor 647	A20	BioLegend	1:50 (10μg/mL)
Goat anti-mouse Granzyme B	N/A	N/A Polyclonal (Cat: AF1865)	R&D Systems	1:100 (2μg/mL)
Rat anti-mouse NKG2D	Biotin	MI-6	Thermo Fisher Scientific	1:50 (10μg/mL)
2° stain				
Streptavidin	Alexa Fluor 594	N/A	BioLegend	1:100 (5μg/mL)
Chicken anti-goat IgG	Alexa Fluor 488	N/A Polyclonal (Cat: A21467)	Thermo Fisher Scientific	1:100 (20μg/mL)
Nuclear counterstain				
DNA	DAPI	N/A	Thermo Fisher Scientific	1ng/mL

Supplementary Table 11. IF panel for detecting granzyme B and NKG2D expression in endogenous and OT-I T cells. Panel used for data in Fig. 3.
