# Supplementary material for: CXCR3 enables recruitment and site-specific bystander activation of memory CD8+ T cells
Source: Nat Commun. 2019 Nov 1;10:4987. doi: 10.1038/s41467-019-12980-2 (PMC6825240; doi:10.1038/s41467-019-12980-2)
Supplement: Supplementary file 3 — Reporting Summary [file 41467_2019_12980_MOESM3_ESM.pdf]

nature research

Corresponding author(s): Martin Prlic
mprlic@fredhutch.org

Last updated by author(s): Oct 8, 2019

Reporting Summary

Nature Research wishes to improve the reproducibility of the work that we publish. This form provides structure for consistency and transparency in reporting. For further information on Nature Research policies, see Authors & Referees and the Editorial Policy Checklist.

Statistics

For all statistical analyses, confirm that the following items are present in the figure legend, table legend, main text, or Methods section.

n/a	Confirmed	
☐	☒	The exact sample size (*n*) for each experimental group/condition, given as a discrete number and unit of measurement
☐	☒	A statement on whether measurements were taken from distinct samples or whether the same sample was measured repeatedly
☐	☒	The statistical test(s) used AND whether they are one- or two-sided<br>*Only common tests should be described solely by name; describe more complex techniques in the Methods section.*
☒	☐	A description of all covariates tested
☒	☐	A description of any assumptions or corrections, such as tests of normality and adjustment for multiple comparisons
☐	☒	A full description of the statistical parameters including central tendency (e.g. means) or other basic estimates (e.g. regression coefficient) AND variation (e.g. standard deviation) or associated estimates of uncertainty (e.g. confidence intervals)
☐	☒	For null hypothesis testing, the test statistic (e.g. *F*, *t*, *r*) with confidence intervals, effect sizes, degrees of freedom and *P* value noted<br>*Give P values as exact values whenever suitable.*
☒	☐	For Bayesian analysis, information on the choice of priors and Markov chain Monte Carlo settings
☒	☐	For hierarchical and complex designs, identification of the appropriate level for tests and full reporting of outcomes
☐	☒	Estimates of effect sizes (e.g. Cohen's *d*, Pearson's *r*), indicating how they were calculated

Our web collection on statistics for biologists contains articles on many of the points above.

Software and code

Policy information about availability of computer code

Data collection	Data were collected using BD FACSDiva, Aperio ImageScope, and Leica SP8 confocal microscope
Data analysis	Flow data were first analyzed using FlowJo v9.6 and v10. Subsequent median fluorescence intensity (MFI) and frequency data were exported into Prism (GraphPad) for statistical analysis.<br>Raw images were imported into HALO (Indica Labs) for cell counting and density calculations. Channels from immunofluorescence images were merged using Adobe Photoshop or ImageJ. When serial sections were used, Adobe Photoshop was used to reorient images from serial sections.

For manuscripts utilizing custom algorithms or software that are central to the research but not yet described in published literature, software must be made available to editors/reviewers. We strongly encourage code deposition in a community repository (e.g. GitHub). See the Nature Research guidelines for submitting code & software for further information.

Data

Policy information about availability of data

All manuscripts must include a data availability statement. This statement should provide the following information, where applicable:

- Accession codes, unique identifiers, or web links for publicly available datasets
- A list of figures that have associated raw data
- A description of any restrictions on data availability

Mouse data will be made available upon request. Deidentified HVTN 908/205 data will be made available with approval from HVTN 908/205 study sponsors.

Field-specific reporting

Please select the one below that is the best fit for your research. If you are not sure, read the appropriate sections before making your selection.

☒ Life sciences ☐ Behavioural & social sciences ☐ Ecological, evolutionary & environmental sciences

For a reference copy of the document with all sections, see nature.com/documents/nr-reporting-summary-flat.pdf

Life sciences study design

All studies must disclose on these points even when the disclosure is negative.

Sample size	Results from initial experiments of N=3 animals (per condition/timepoint) were used to determine subsequent N values needed to sufficiently power experiments.
Data exclusions	Data from mice which were unsuccessfully infected were excluded. Infection success was validated via immunofluorescence and colony forming unit assays performed on spleen and liver.
Replication	Technical replicates were performed for all mouse studies (Fig 2-7). All replicates were successful.<br><br>Technical replicates using biologics (NKG2D blocking antibody, clone: HMG2D, vendor: BioXCell, Cat: BE0111) (data not shown), were not successful. Subsequent immune responses varied on a lot-to-lot basis.<br><br>Technical replicates for HVTN205/908 data (Fig 1, Fig S1) were not conducted due to extremely limited sample availability from early time points in vaccine studies.
Randomization	In HVTN 908/205, human participants were randomized to placebo or vaccination in a double-blinded manner.<br><br>In mouse studies, we did not randomize mice to infection, timepoint, or treatment. Instead, we matched animals to have similar baseline (pre LM infection) memory OT-I cell frequencies in blood. We did this to minimize any differences in bystander activation or cell density that may result from higher or lower OT-I frequency.
Blinding	HVTN 908/205 samples were blinded until analysis was complete. Afterwards, researchers were unblinded to whether participants had been given MVA/HIV62 or saline placebo.<br><br>Researchers were not blinded to infection status of mice, as uninfected controls and infected animals were housed in separate cages. Flow (.fcs) files were assigned metadata indicating mouse identity, infection status, time points, and tissue types. Blinding was not necessary as gating calls (for flow cytometry) and automated positivity calls (for immunofluorescence) were set on negative (uninfected) controls.

Reporting for specific materials, systems and methods

We require information from authors about some types of materials, experimental systems and methods used in many studies. Here, indicate whether each material, system or method listed is relevant to your study. If you are not sure if a list item applies to your research, read the appropriate section before selecting a response.

Materials & experimental systems

n/a	Involved in the study	
☐	☒	Antibodies
☒	☐	Eukaryotic cell lines
☒	☐	Palaeontology
☐	☒	Animals and other organisms
☐	☒	Human research participants
☐	☒	Clinical data

Methods

n/a	Involved in the study	
☒	☐	ChIP-seq
☐	☒	Flow cytometry
☒	☐	MRI-based neuroimaging

Antibodies

Antibodies used	BUV395 mouse anti-mouse CD45.1 (clone A20) BD Biosciences Cat# 565212, RRID: AB_2722493<br><br>BUV737 rat anti-mouse IFNγ (clone XMG1.2) BD Biosciences Cat# 564693, RRID: AB_2722494<br><br>BV421 hamster anti-mouse CD183 (CXCR3) (clone CXCR3-173) BD Biosciences Cat# 562937, RRID: AB_2687551<br><br>BV605 hamster anti-mouse KLRG1 (clone 2F1) BD Biosciences Cat# 564013, RRID: AB_2722497<br><br>BV711 rat anti-mouse CD314 (NKG2D) (clone CX5) BD Biosciences Cat# 563694, RRID: AB_2722498

BV786 rat anti-mouse CD90.1 (Thy1.1) (clone OX-7) BD Biosciences Cat# 740917

PE-CF594 rat anti-mouse CD8α (clone 53-6.7) BD Biosciences Cat# 562283, RRID: AB_11152075

BUV395 mouse anti-human CD8 (clone RPA-T8) BD Biosciences Cat# 563795, RRID: AB_2722501

BUV661 mouse anti-human HLA-DR (clone G46-6) BD Biosciences Cat# 565073, RRID: AB_2722500

BUV737 mouse anti-human CD69 (clone FN50) BD Biosciences Cat# 564439, RRID: AB_2722502

BV786 mouse anti-human CD3 (clone SK7) BD Biosciences Cat# 563800

BV421 mouse anti-human CD137 (4-1BB) (clone 4B4-1) BD Biosciences Cat# 564091, RRID: AB_2722503

BV510 mouse anti-human CD45RA (clone HI100) BD Biosciences Cat# 563031, RRID: AB_2722499

BB700 mouse anti-human CD56 (clone B159) BD Biosciences Cat# 566400, RRID: AB_2722504

PE-CF594 mouse anti-human CD197 (CCR7) (clone 150503) BD Biosciences Cat# 562381, RRID: AB_11153301

PE-Cy7 mouse anti-human CD279 (PD-1) (clone EH12.1) BD Biosciences Cat# 561272, RRID: AB_10611585

Alexa Fluor 700 mouse anti-human/mouse Granzyme B (clone GB11) BD Biosciences Cat# 560213, RRID: AB_1645453

APC-H7 mouse anti-human CD45RO (clone UCHL1) BD Biosciences Cat# 561137, RRID: AB_10562194

PE rat IgG2a isotype control BD Biosciences Cat# 554689, RRID: AB_479724

DyLight 488 donkey anti-rabbit IgG (clone Poly4064) BioLegend Cat# 406404, RRID: AB_1575130

Alexa Fluor 594 rat anti-mouse CD169 (Siglec-1) (clone 3D6.112) BioLegend Cat# 142416, RRID: AB_2565620

PE-Cy7 rat anti-mouse Ki-67 (clone 16A8) BioLegend Cat# 652426, RRID: AB_2632694

Purified rat anti-human/mouse Ki-67 (clone 11F6) BioLegend Cat# 151202, RRID: AB_2566621

BV605 mouse anti-human CD161 (clone HP-3G10) BioLegend Cat# 339916, RRID: AB_2563607

BV711 mouse anti-human CD4 (clone OKT4) BioLegend Cat# 317440, RRID: AB_2562912

FITC mouse anti-human TCR Valpha7.2 (clone 3C10) BioLegend Cat# 351704, RRID: AB_10900975

Purified goat anti-mouse Granzyme B (polyclonal) R&D Systems Cat# AF1865, RRID: AB_2294988

PE rat anti-mouse Rae-1 (pan-specific) (clone 186107) R&D Systems Cat# FAB17582P, RRID:AB_357086

eFluor 450 rat anti-mouse IL-2 (clone JES6-5H4) Thermo Fisher Scientific Cat# 48-7021-82, RRID: AB_1944462

FITC rat anti-mouse CD44 (clone IM7) Thermo Fisher Scientific Cat# 11-0441-85, RRID: AB_465046

FITC rat anti-mouse CD62L (L-selectin) (clone MEL-14) Thermo Fisher Scientific Cat# 11-0621-85, RRID: AB_465110

Alexa Fluor 488 donkey anti-goat IgG Thermo Fisher Scientific Cat# A-11055, RRID: AB_2534102

PerCP-Cy5.5 rat anti-mouse CD90.2 (Thy1.2) (clone 30-H12) Thermo Fisher Scientific Cat# 46-0903-82, RRID: AB_10670882

PE hamster anti-mouse CD314 (NKG2D) (clone A10) Thermo Fisher Scientific Cat# 12-5872-82, RRID: AB_465980

PE mouse anti-human/mouse Granzyme B (clone GB11) Thermo Fisher Scientific Cat# GRB04, RRID: AB_2536538

PE-Cy7 mouse anti-mouse Granzyme A (clone GzA-3G8.5) Thermo Fisher Scientific Cat# 25-5831-82, RRID: AB_2573476

APC rat anti-mouse CD8β (clone eBioH35-17.2) Thermo Fisher Scientific Cat# 17-0083-81, RRID: AB_657760

Alexa Fluor 647 chicken anti-rat IgG Thermo Fisher Scientific Cat# A-21472, RRID:AB_2535875

APC-eFluor 780 rat anti-mouse CD127 (clone A7R34) Thermo Fisher Scientific Cat# 47-1271-82, RRID: AB_1724012

Biotin mouse anti-mouse CD45.1 (clone A20) Thermo Fisher Scientific Cat# 13-0453-85, RRID: AB_466454

Biotin rat anti-mouse CD90.1 (clone HIS51) Thermo Fisher Scientific Cat# 13-0900-85

Alexa Fluor 488 rat anti-mouse CD90.1 (clone OX-7) BioLegend Cat# 202506

Rabbit Listeria O Antiserum Poly Serotypes 1, 4 (polyclonal) Difco, Becton Dickenson Cat# 223021

PE GILGFVFTL (IAV M158-66) HLA-A*02 tetramer Fred Hutch: Immune Monitoring Core https://sharedresources.fredhutch.org

APC YVLDHLIVV (EBV BRLF1190-198) HLA-A*02 tetramer Fred Hutch: Immune Monitoring Core https://sharedresources.fredhutch.org

Validation	All flow cytometry antibody performance was validated by titration and fluorescence minus ones.<br>CXCR3 and NKG2D antibody performance was further validated in knockout mice.<br>Congenic markers (CD45.1 and Thy1.1) were validated in mice with and without congenic markers and/or adoptive transfers for both flow cytometry and immunofluorescence.<br>LM antisera used for detection in immunofluorescence was tested in uninfected and infected animals. Further use is described in Shi C, et al (2011) Journal of Immunology.

Animals and other organisms

Policy information about studies involving animals; ARRIVE guidelines recommended for reporting animal research

Laboratory animals	All animal studies used female mice. Of these, C57BL/6J, C57BL/6-Tg(TcraTcrb)1100Mjb/J SJL (OT-I SJL), CXCR3 knockout OT-Is (B6.129P2-Cxcr3tm1Dgen/J (CXCR3 knockouts) crossed with C57BL/6-Tg(TcraTcrb)1100Mjb/J SJL, and backcrossed to OT-I), and PL P14 mice expressing DbGP33-specific TCR backcrossed to C57BL/6.<br>Mice used for adoptive transfers were 8-40 weeks of age.<br>Mice used as cell recipients were 6-10 weeks of age at adoptive transfer. Cell recipient mice were sacrificed at 15-40 weeks of age.
Wild animals	Study did not involve wild animals.
Field-collected samples	Study did not involve samples collected from the field.
Ethics oversight	The Fred Hutchinson Cancer Research Center Institutional Animal Care and Use Committee provided guidance and approved all mouse protocols used for this study.

Note that full information on the approval of the study protocol must also be provided in the manuscript.

Human research participants

Policy information about studies involving human research participants

Population characteristics	HVTN 908/205 participants were 18-50 years of age, were male or female, of low risk for HIV infection, and not previously vaccinated with MVA.<br><br>HVTN 908/205 participants were healthy (via medical history, physical examination, laboratory tests, troponin levels, and electrocardiogram) at enrollment. Participants were adults aged 18-50 years, of male or female sex, considered at low risk for HIV infection, and not previously vaccinated with MVA. The protocol was conducted in a randomized, double-blind, placebo control trial.
Recruitment	*Describe how participants were recruited. Outline any potential self-selection bias or other biases that may be present and how these are likely to impact results.*
Ethics oversight	HIV Vaccine Trials Network Institutional Review Boards

Note that full information on the approval of the study protocol must also be provided in the manuscript.

Clinical data

Policy information about clinical studies
All manuscripts should comply with the ICMJE guidelines for publication of clinical research and a completed CONSORT checklist must be included with all submissions.

Clinical trial registration	ClinicalTrials.gov NCT00908323, NCT00820846
Study protocol	https://clinicaltrials.gov/ct2/show/NCT00820846<br>https://clinicaltrials.gov/ct2/show/NCT00908323
Data collection	HVTN and HVTN partner sites in the US (Alabama, California, Washington state) and Peru. Recruitment began in July 2009 and ran until July 2012. Data collection from clinical samples for this study (Fig 1, Fig S1) was conducted December 2017.
Outcomes	*Describe how you pre-defined primary and secondary outcome measures and how you assessed these measures.*

Flow Cytometry

Plots

Confirm that:

☒ The axis labels state the marker and fluorochrome used (e.g. CD4-FITC).

☒ The axis scales are clearly visible. Include numbers along axes only for bottom left plot of group (a 'group' is an analysis of identical markers).

☒ All plots are contour plots with outliers or pseudocolor plots.

☒ A numerical value for number of cells or percentage (with statistics) is provided.

Methodology

Sample preparation

Human PBMC samples from HVTN205/908 were retrieved from long-term storage in the vapor phase of liquid nitrogen, thawed in a 37°C water bath, and resuspended in RPMI supplemented with 10% FBS, L-Glutamine, and Penicillin/Streptomycin. PBMC were washed and transfered to 96-well round-bottom plates for flow staining. Viability staining and Fc blocking was conducted in 1x PBS for 20' at room temperature. Tetramer staining was conducted in FACSWash (1x PBS supplemented with 2% FBS) for 1 hour at room temperature. Surface staining was conducted in FACSWash for 20' at room temperature. Cells were fixed with BD Cytofix/Cytoperm for 20' at room temperature. Intracellular staining was conducted in 1x BD Perm/Wash buffer for 30' at room temperature.

Peripheral blood was acquired from mice prior to sacrifice via facial vein puncture, and collected in tubes containing heparin. Peripheral blood was lysed in 1mL ACK lysis buffer for 3'. Lymph nodes and spleens were collected post sacrifice in ice-cold RP10 (RPMI supplemented with 10% FBS, HEPES, Sodium Pyruvate, L-Glutamine, and Penicillin/Streptomycin). Lymph nodes and spleens were mechanically dissociated through a 70µm filter in ice-cold RP10. Contaminating splenic erythrocytes were lysed by incubating splenocytes in a 50/50 mixture of RP10 and ACK lysis buffer for 20'. Cells were transferred to 96-well plates for flow cytometry staining. Viability staining was conducted in 1x PBS for 20' on ice. Surface marker staining and Fc blocking was conducted in FACSWash (supplemented with 0.02% sodium azide) for 30' on ice. Cells were fixed in BD Cytofix/Cytoperm buffer for 20' on ice. Intracellular marker staining was conducted in 1x BD Perm/Wash for 30' on ice.

Instrument

We used a BD LSR-II and BD FACSSymphony equipped with UV, violet, blue, green, and red lasers.

Software

We used FlowJo v9 and v10 (BD) to analyze data.

Cell population abundance

We enriched for TCR-transgenic CD8+ T cells (wildtype OT-I, CXCR3-/- OT-I, and wildtype P14) using magnetic-activated cell sorting (MACS). We assessed post MACS purity using flow cytometry on pre- and post-sort fractions, staining for TCR Vbeta5 (for OT-I), CD4, CD8a, CXCR3, and congenic markers (CD45.1, CD45.2 for OT-I, Thy1.1, Thy1.2 for P14). Post-MACS purity of CD8+ T cells was >99%.

Gating strategy

For mouse experiments, all events were first time gated, excluding events collected during moments of unstable fluidics. From time-gated events, we gated singlets from FSC-H and -A profile (in which events with a high FSC-A but dissimilarly high FSC-H were excluded). From singlets, we gated live events, which were low for amine-reactive viability dye. From live events, we gated lymphocytes based off of FSC/SSC profile, excluding events with higher FSC and SSC (e.g. monocytes, granulocytes) and events with very low FSC and SSC (e.g. debris). From lymphocyte events, we gated CD8a+ cells which separates into discrete populations. From CD8a+ events, we excluded CD8aa cells (CD45.1+ OT-I cells, which are solely CD8ab were used to identify endogenous CD8aa cells). From CD8ab cells, we gated splenic cells from the red pulp and white pulp via i.v. CD8b labeling. RP events were gated based off of blood from the same mouse (which is uniformly labeled). WP events were gated based off of falling outside of the CD8b gate and having a defined population (i.e. intermediate events were excluded). From RP and WP events, we gated OT-I events (CD45.1+) and endogenous CD8 events (CD45.1-).

For human experiments, all events were first time gated, excluding events collected during moments of unstable fluidics. From time-gated events, we gated singlets from FSC-H and -A profile (in which events with a high FSC-A but dissimilarly high FSC-H were excluded). From singlets, we gated live events, which were low for amine-reactive viability dye. From live events, we gated lymphocytes based off of FSC/SSC profile, excluding events with higher FSC and SSC (e.g. monocytes, granulocytes) and events with very low FSC and SSC (e.g. debris). From lymphocyte events, we excluded APC (CD3- HLA-DR-) events. From non APC lymphocytes, we gated NK cells (CD56+ CD3-) and T cells (CD3+) events. From CD3+ T cells, we gated CD4+ and CD8+ and double negative (DN, CD4- CD8-) T cells. From CD8+ and DN T cell events, we gated MAIT cells (CD161+ TCRValpha7.2+). From CD4+ and CD8+ T cells we gated memory (CD45RO+) and naïve (CD45RO-) cells. From memory, naïve, MAIT, and NK cell subsets, we gated CCR7, CD45RA, Granzyme B, 4-1BB, HLA-DR, CD69, and PD-1.

☒ Tick this box to confirm that a figure exemplifying the gating strategy is provided in the Supplementary Information.
